# Combined Metabolome and Transcriptome Analysis of Floral Organ Development in *Magnolia cavaleriei* var. *platypetala* ‘Tanchun’

**DOI:** 10.3390/plants15111646

**Published:** 2026-05-27

**Authors:** Yingbing Hu, Zhe Zhang, Yubing Yong, Minhuan Zhang, Xijun Hu, Weiqun Lei, Donglin Zhang

**Affiliations:** 1 College of Landscape Architecture, Central South University of Forestry and Technology, Changsha 410004, China; 20230100084@csuft.edu.cn (Y.H.); zzxy.la@gmail.com (Z.Z.); t20202505@csuft.edu.cn (Y.Y.); t20040180@csuft.edu.cn (M.Z.); t20040191@csuft.edu.cn (X.H.); 2 College of Urban and Rural Construction, Zhejiang Guangsha Vocational and Technical University of Construction, Dongyang 322100, China; 3 College of Arts, Nanning University, Nanning 530200, China; 4 Department of Horticulture, University of Georgia, Athens, GA 30602, USA

**Keywords:** *Magnolia*, *Michelia cavaleriei*, floral scent, aroma development, VOC composition, flower development, floral organs

## Abstract

*Magnolia cavaleriei* var. *platypetala* ‘Tanchun’ is a newly registered flower variety in China, known for its characteristic floral aroma that intensifies toward full bloom. However, the composition of the volatiles of this aromatic flower remains uncharacterized. Here, we compared the volatile organic compound composition of Tanchun through gas chromatography–mass spectrometry and comparative transcriptome sequencing analyses of the stamen (S), pistil (P), and petals (T) during flower development, i.e., the bud (S1), semi-opened (S2), and bloom (S3) stages. We present a first comprehensive profile of 1395 metabolites from Tanchun’s floral organs. Terpenoids (26.2%) constituted the largest chemical group, followed by esters (17.52%), nitrogen compounds (9.83%), hydrocarbons (8.11%), alcohols (7.97%), aldehydes (6.53%), and others. We found that volatile organic compound (VOC) accumulation was both spatiotemporal and stage-specific. The S1 and S2 transition was characterized by scent notes of green, herbal, and waxy aromas, while the S2 and S3 shift exhibited a richer profile of fruity, sweet, and creamy notes, primarily in petals. A comparative VOC and transcriptomic analysis revealed that petals activate pathways for structural expansion and precursor mobilization, stamens enhance lipid and terpenoid metabolism, and pistils maintain a conserved profile. Importantly, the S1 and S2 transition in petals establishes the biochemical foundation by activating acyl-CoA, phenylpropanoid, and terpenoid synthesis pathways, which enables the activation of the butanoate metabolism pathway at S3, leading to the production of ester-rich compounds that define the floral scent. The transition to full bloom involves a shift to energy-efficient volatile biosynthesis, supported by carbohydrate restructuring and phytohormonal regulation. Our results provide the first comprehensive volatilome and transcriptome resource for ‘Tanchun’, revealing a highly efficient, multi-stage strategy for floral fragrance biosynthesis. This work lays a molecular foundation for future horticultural improvement and biotechnological applications in the flavor and fragrance industries.

## 1. Introduction

Floral scent is a key mediator of plant–pollinator communication and reproductive success. It provides species-specific chemical cues that guide pollinator attraction, recognition, and behavior [1]. These volatile organic compounds (VOCs) originate mainly from terpenoid, phenylpropanoid/benzenoid, and fatty acid-derived pathways, and their emission patterns change dynamically across floral development [2,3]. Many flowering species exhibit a coordinated shift from early structural growth to a late, metabolically specialized phase that supports peak scent emission at anthesis [4,5]. Such developmental modulation is prominent in species with strong aromas, where precise timing of VOC production is essential for pollinator interaction.

The biosynthesis of floral volatiles is governed by a complex network of metabolites. These involve activation of precursors, hydrolysis of glycosides, interconversion of aldehydes/alcohols, and conjugation/deconjugation of volatiles [6,7]. Enzymes, including *β*-glucosidases (BGLs), alcohol acyltransferases (AATs), O-methyltransferases (OMTs), and terpene synthases (TPSs), play central roles in generating scent profiles [8]. Recent results have drawn attention to important metabolism-related pathways as important contributors to short-chain esters with fruity and sweet aroma notes in flowers [9,10]. The key steps in this pathway are common in fruit aroma biosynthesis, which integrates transamination of branched-chain amino acids, oxidation of aldehyde, formation of acyl-CoA, and the use of AAT enzymes for final esterification [11,12]. Its regulation is coordinated by transcription factors, including bHLH/MYC2, ERF, WRKY, and MYB families, which modulate floral metabolic flux in response to jasmonate and ethylene signaling [13]. Thus, floral aroma is established as an output of a precisely orchestrated network of metabolic pathways, under the influence of complex transcriptional regulation, related to VOC biosynthesis.

Multi-omics strategies have considerably enhanced our perception of floral scent by involving transcriptome variations with accumulation patterns of metabolites. Integration of metabolome–transcriptome in rose [14], *Jasminum sambac* [15], *Prunus mume* [16], *Paeonia suffruticosa* Andr [17], and *Nelumbo nucifera* [18] revealed stage-specific reprogramming of glycoside hydrolysis, phenylpropanoid turnover, and ester or terpene biosynthesis. These studies also highlighted the importance of AGLs and glycosyltransferases in releasing glycosidically bound precursors that contribute substantially to floral scent complexity [19]. Though such studies are abundant in aromatic plant species, such knowledge is limited for the species of *Magnolia*, a genus consisting of more than 200 species. Most research in this genus has been dedicated to work on the volatile compounds and characteristic order of essential oils from species such as *Magnolia obovata* (leaves) [20], *Magnolia grandiflora* (flowers) [21], *Magnolia pugana* (leaves, flowers, and seeds) [22], the flower buds of *Magnolia heptapeta* and *Magnolia denudata* var. purpurascens [23], and others. Though these studies provide fundamental information on the composition of VOCs in the flowers of the genus *Magnolia,* specific species of interest still need to be studied for their organ-specific VOCs.

*Magnolia cavaleriei* (previously known as *Michaelia cavaleriei*) is one of the least-explored species of *Magnolia* in regard to its scent and aroma profiles. *M. cavaleriei* plants grow to a maximum height of 10 m with silver–grey buds, narrowly oblanceolate–oblong or narrowly oblong leaves with an acuminate leaf apex and a cuneate or broadly cuneate leaf base, and 10–12 obovate-elliptic tepals; the plants flower in March and bear fruit in September–October [24]. This species is native to southern and south-central China, where it is found in forests at elevations between 800 and 2400 m (http://www.eFloras.org/; accessed on 19 November 2025). Within China, its native range is widespread across Fujian, Guangdong, Guangxi, Guizhou, Hubei, Hunan, Sichuan, and Yunnan. Particularly, the region including Yunnan, Guizhou, and Guangxi is considered the hotspot for the wild *Magnoliaceae* species in China [25]. Recently, a new variety called Tanchun (*M. cavaleriei* var. *platypetala*) has been granted a plant variety right (variety right number: 20250312) by the China Forestry and Grassland Administration in July 2025 [26]. Tanchun produces a characteristic floral aroma that intensifies toward full bloom (authors’ observations), yet the composition of the VOCs and relevant transcriptional regulation underlying this aromatic profile are unknown. To address this gap, we performed an integrated metabolome and transcriptome analysis across three floral developmental stages of three floral organs (petals, stamens, and pistils). By identifying differentially expressed genes (DEGs), enriched pathways, and stage-specific metabolic signatures, we aimed to provide a detailed list of VOCs and related transcriptional changes governing aroma formation in relation to organs and growth stage. The results of this study provide a mechanistic framework for understanding floral scent biosynthesis in *M. cavaleriei* var. *platypetala* ‘Tanchun’, identifying petals as the key organ regulating metabolite accumulation and volatile release at anthesis.

## 2. Results

### 2.1. Global Metabolome Analysis

**Figure 1:**
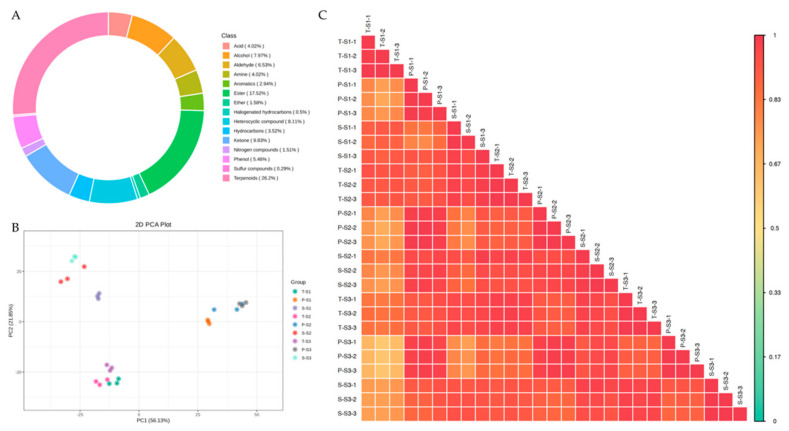
Global VOC profiles of *M. cavaleriei* floral organs at different growth stages. (**A**) VOC classes and their %; (**B**) principal component analysis; and (**C**) Pearson correlation coefficient analysis on the basis of relative metabolite intensity. T = petals, P = pistils, and S = stamens. The suffixes −1, −2, and −3 represent three biological replicates (i.e., independent samples) of each tissue and developmental stage. S1 = bud stage, S2 = initial flowering stage, and S3 = full flowering stage.

We identified the main VOCs in *M. cavaleriei* at three distinct flower blossom stages (S1, S2, and S3) among flower parts (P, S, and T). A total of 1394 metabolites belonging to 15 chemical classes were detected; terpenoids (26.2%) constituted the largest group, followed by esters (17.52%), nitrogen compounds (9.83%), hydrocarbons (8.11%), alcohols (7.97%), aldehydes (6.53%), and others (Figure 1A; Supplementary Table S1). Principal component analysis revealed that PC1 and PC2 accounted for 56.13% and 21.85% of the variance, respectively (Figure 1B). The clear grouping of the samples suggests distinct differences in metabolite composition among different flower organs and developmental stages. The Pearson correlation coefficient analysis indicated higher correlation among biological replicates (Figure 1C). The total VOC intensity decreased progressively from S1 (49,470.12) to S3 (39,751.20), indicating stronger volatile release at early flowering. Among organs, petals showed the highest VOC levels, peaking at T-S1 (18,816.81) and declining by T-S3 (14,291.34). Pistils and stamens also followed a similar downward trend, though stamens showed a slight rise at S2. Overall, VOC emission was both stage- and organ-dependent, with petals contributing most prominently during early bloom.

### 2.2. Composition Among Flower Organs (Petals, Stamens, and Pistils) at Three Developmental Stages of the Flower

**Figure 2:**
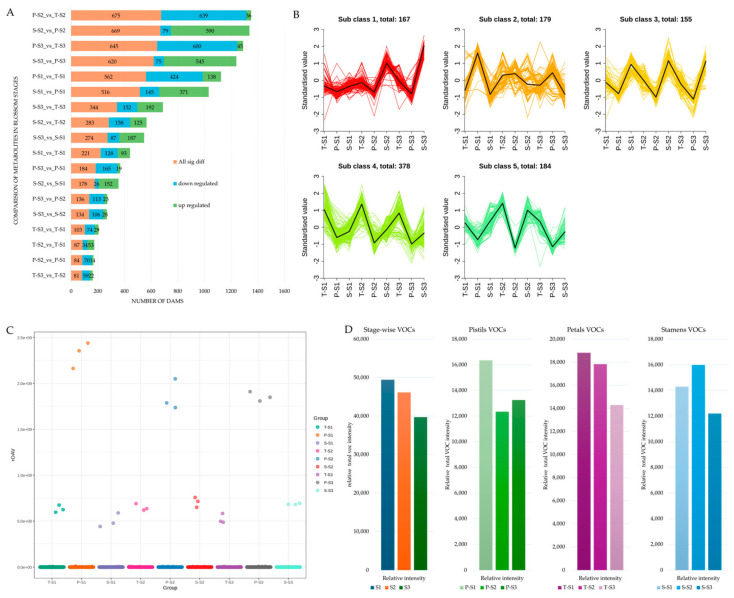
Comparative analysis of DAMs. (**A**) DAMs in pairwise comparisons across three development stages among three organs. (**B**) K-means clustering of differential metabolites. The horizontal axis represents sample grouping, the vertical axis represents the standardized relative content of metabolites, and the subclass represents the category number of metabolites with the same trend of change. Total: The number of metabolites representing this category. (**C**) Relative odor activity value (rOAV) distribution of volatile metabolites across different flower organs and developmental stages. (**D**) Total volatile organic compound (VOC) intensity across flowering stages and floral organs. T = petals, P = pistils, and S = stamens. The suffixes −1, −2, and −3 represent three biological replicates (i.e., independent samples) of each tissue and developmental stage. S1 = bud stage, S2 = initial flowering stage, and S3 = full flowering stage.

The number of differentially accumulated metabolites (DAMs) among the three floral organs across the three developmental stages revealed clear organ- and stage-specific metabolic patterns. A large number of significant DAMs were detected in pairwise comparisons, reflecting dynamic changes in floral volatile biosynthesis throughout flower development. Across all comparisons, both the petals and stamens showed strong metabolic activity, while pistils displayed fewer differential changes, suggesting more stable metabolism. Comparisons such as S-S3 vs. P-S3 (545 up- and 75 downregulated) and S-S2 vs. P-S2 (590 up- and 79 downregulated) showed a large number of upregulated metabolites in stamens, highlighting their prominent role in synthesizing and emitting floral volatiles during flower opening. Petals also exhibited substantial differences, particularly in P-S1 vs. T-S1 (138 up- and 424 downregulated) and P-S3 vs. T-S3 (600 up- and 45 downregulated), where numerous compounds were DAMs, confirming that petals contribute significantly to floral scent emission and regulation throughout development. The pistils, although less variable, appeared to maintain certain key volatiles, possibly linked to reproductive function. Within each organ, clear stage-dependent metabolic shifts were observed (Figure 2A). The majority of DAMs in pistils in P-S2 vs. P-S1, P-S3 vs. P-S1, and P-S3 vs. P-S2 showed reduced accumulations, suggesting a gradual reduction in some volatiles as the flower matured. In contrast, stamens displayed more active regulation, with 178 (S-S2 vs. S-S1), 274 (S-S3 vs. S-S1), and 134 (S-S3 vs. S-S2) differential metabolites, with a substantial number being upregulated, indicating increased synthesis of aroma-related compounds during blooming. Petals also showed moderate but consistent stage-specific changes, with 87, 103, and 81 differential metabolites in T-S2 vs. T-S1, T-S3 vs. T-S1, and T-S3 vs. T-S2, respectively. These results demonstrate that metabolite expression in Tanchun flowers is both organ- and stage-specific.

The K-means clustering analysis delineated five distinct volatile signatures (K1–K5) that revealed a highly dynamic and organ-specific regulation of the floral volatilome across development (Figure 2B). During S1, the K4 cluster was prominent, including hydrocarbons, acids, amines, and terpenoids, suggesting an emphasis on foundational lipid metabolism and potential protein breakdown (Supplementary Table S2). The transition from S1 to S2 was characterized by the K2 cluster, indicating an alcohol, esters, and terpenoids-rich profile. This indicates that at the S2 stage, the flowers synthesize odor-related compounds. Further, during the transition to the S3 stage, the VOC profile diversified. While the pistils and stamens were characterized by a terpenoid-rich signature (K5 and K3), the petals exhibited a relatively complex mixture of esters (K1), alcohols (K3), and an increase in nitrogen/sulfur compounds. These observations suggest a shift toward a somewhat consistent VOC profile alongside the early onset of senescence. These sequential changes in VOCs highlight a precise temporal and spatial synthesis and accumulation, which is well aligned with the flower’s reproductive growth and development.

### 2.3. rOAV Profiling of Metabolites Among Three Organs in Three Developmental Stages

The rOAV analysis (rOAV value ≥ 1 = 402 VOCs) revealed distinct variations in the contribution of floral volatile compounds (Figure 2C; Supplementary Table S3), including terpenoids (88), esters (74), aldehydes (51), alcohols (40), ketones (38), phenols (38), heterocyclic compounds (32), aromatics (17), and others (24). At the bud stage (S1), pistils and stamens exhibited the highest rOAVs, suggesting early accumulation of key aroma-active metabolites. During the initial opening stage (S2), rOAVs increased in petals and pistils, indicating enhanced volatile release as the flowers began to open. In the full-bloom stage (S3), petals and stamens maintained moderate to high rOAVs, reflecting the establishment of a mature floral scent profile. The overall VOC intensity declined from S3 to S1, indicating a decrease in volatile emission during flower development. Among floral organs, petals exhibited the highest total VOC intensity at all stages, followed by pistils and stamens (Figure 2D). This pattern highlights that volatile production is both stage- and organ-dependent, with early-stage petals being the major contributors to floral scent.

### 2.4. Identification of Characteristic Floral Components in Tanchun

**Figure 3:**
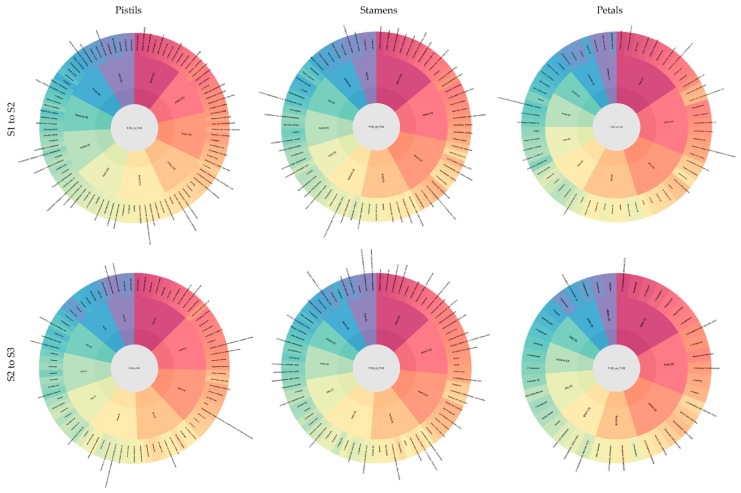
Differential metabolite sensory wheels. The innermost circle represents the differential comparison group, the second circle represents the top 10 sensory flavor features with the highest number of annotated differential metabolites in the comparison group, the numbers in parentheses indicate the number of differential metabolites annotated for that sensory flavor feature, and the outermost circle represents the differential metabolites. If the number of differential metabolites annotated for a certain sensory flavor feature exceeds 10, the top 10 differential metabolites with the highest VIP value are displayed. Different colors in the second circle represent different sensory flavor categories, and the same color corresponds to the same sensory category across all panels.

The most significant VOCs are necessarily required for further screening among the key biomarkers to distinguish the organ/stage. In this regard, we compared the 402 compounds exhibiting rOAV ≥ 1 (Supplementary Table S3) with DAMs (VIP >1, FC| ≥ 2, and ≤0.5; Supplementary Table S4), and identified 325 VOCs. These VOCs also represent different groups of K-means clustering, especially the K2 and K4 VOCs (Supplementary Table S5). Pistils were characterized by fruity, sweet, minty, floral, fresh, herbal, balsamic, musty, waxy, citrus, fresh, fatty, herbal, floral, and green notes (Figure 3). Stamens had phenol, fatty, rose, sweet, fresh, fruity, balsamic, creamy, waxy, herbal, and green notes. Interestingly, petals, in addition to these notes, were characterized by coconut, oily, alkane, cinnamon, and cucumber notes. These observations suggest that though common notes are present in the three organs, petals have characteristic notes. Thus, our results highlight that the petals are the major organs for VOCs that are related to aroma and flavor in Tanchun. Sunburst visualization of volatile compounds across petal developmental stages (S1–S3) revealed clear stage-specific patterns in metabolite composition. Terpenoids and esters were dominant in mature petals (S3), contributing most to the floral aroma, while alcohols and aldehydes were relatively higher during early and intermediate stages (S1 and S2). These trends indicate a progressive enhancement of scent-related metabolites as the flowers mature.

During floral development, the petals exhibited marked stage-specific variation in volatile metabolite accumulation (Table 1, Supplementary Table S4). In petals, during the transition from S1 to S2 (T_S2_vs_T_S1), several compounds showed significant upregulation, including cyclohexanol, 1-methyl-4-(1-methylethenyl)-(a terpenoid with woody and fresh notes), methyl tiglate (a fruity ester), and benzene–emethanol, 3,4-dimethoxy- (a sweet aromatic alcohol), indicating enhanced floral and fruity scent formation. In the S2 to S3 transition (T_S3_vs_T_S2), increases in phenol, o-amino-, 2,6-octadien-1-ol, 3,7-dimethyl- (Z)-, and ascaridole reflected enrichment of phenolic and terpenoid volatiles, contributing to the mature floral and slightly spicy aroma at late flowering. When comparing S3 to S1 (T_S3_vs_T_S1), consistently elevated levels of acetonitrile, 2,2′-iminobis-, cyclohexanol, 1-methyl-4-(1-methylethenyl)-, 2,6-octadien-1-ol, 3,7-dimethyl-(Z)-, and esters such as phenylacetic acid propyl ester and butanoic acid, 2-methylphenyl ester highlighted the progressive enhancement of nitrogenous, terpenoid, and ester-derived volatiles. These results suggest that petal maturation is accompanied by dynamic biosynthesis of fragrance-related compounds, particularly terpenoids, esters, and aromatic alcohols, which shape the characteristic floral VOCs (and aroma) of Tanchun.

### 2.5. KEGG Pathway Enrichment of DAMs

**Figure 4:**
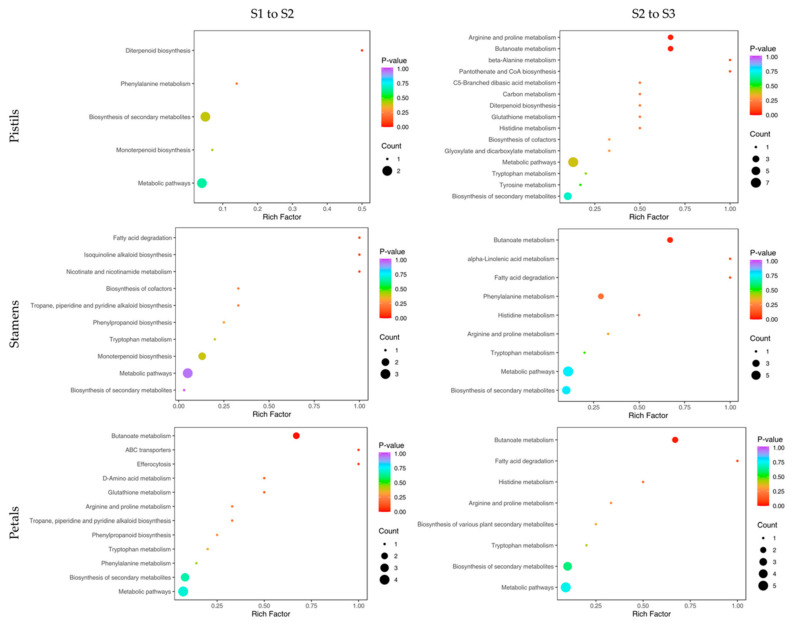
Enrichment of DAMs in KEGG pathways. The horizontal axis represents the Rich Factor for each pathway, and the vertical axis represents the pathway name (sorted by *p*-value). The color of the dots reflects the *p*-value, with redder dots indicating more significant enrichment. The size of the dots represents the number of differentially enriched metabolites. S1 = bud stage, S2 = initial flowering stage, and S3 = full flowering stage.

The DAMs were significantly enriched in a range of secondary metabolism pathways. The DAMs in pistils (S1 to S2) were enriched in mono- and di-terpenoid, secondary metabolite biosynthesis, and phenylalanine metabolism pathways, whereas the DAMs in S2 to S3 were enriched in relatively higher numbers of pathways related to amino acid (arginine, proline, beta-alanine, glutathione, tryptophan, and tyrosine), butanone, carbon, diterpenoids, glyoxylate, and dicarboxylate metabolism pathways. These changes, consistent with the accumulation trends, indicate that several pathways are activated in a stage-specific manner. In the case of stamens, DAMs in transition from S1 to S2 were enriched in pathways related to fatty acid degradation; isoquinoline alkaloid biosynthesis; monoterpenoid; tropane, piperidine, and pyridine alkaloids; nicotinate and nicotinamide; and tryptophan metabolism. Whereas the DAMs in S2 to S3 in stamens were associated with pathways related to butanoate, alpha-linolenic acid, phenylalanine, histidine, arginine, proline, tryptophan metabolism, and fatty acid degradation. Most importantly, in the T_S2 vs. T_S1 comparison, enrichment of butanoate metabolism, arginine and proline metabolism, and phenylpropanoid biosynthesis suggested activation of primary amino acid and aromatic compound metabolism contributing to early scent development. The T_S3 vs. T_S2 stage showed strong enrichment in fatty acid degradation, tryptophan metabolism, and secondary metabolite biosynthesis, indicating enhanced production of volatile precursors and defense-related compounds during floral maturation. Meanwhile, the T_S3 vs. T_S1 comparison highlighted broad enrichment in glutathione metabolism, pantothenate and CoA biosynthesis, and phenylpropanoid biosynthesis, reflecting increased redox balance, cofactor synthesis, and aromatic secondary metabolism in fully open flowers (Figure 4). Collectively, these enrichment results highlight the stage-specific accumulation of terpenoids, esters, and phenylpropanoids, which contribute to the distinctive aroma profile of Tanchun petals (Figure 3).

### 2.6. Transcriptome Sequencing of M. cavaleriei

#### 2.6.1. Global Transcriptome Profiles of *M. cavaleriei* Floral Organs During Development

**Figure 5:**
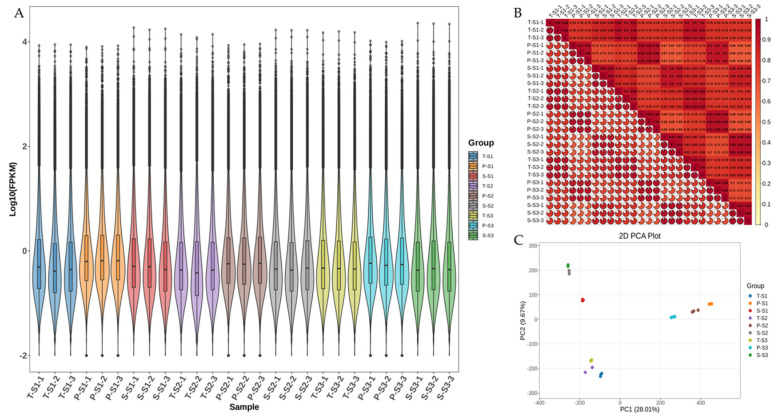
Global transcriptome profile of Tanchun floral organs at different growth stages. (**A**) Overall gene expression; (**B**) Pearson’s correlation coefficient analysis comparison between samples and replicates; (**C**) principal component analysis of gene expression in different organs at different growth stages. T = petals, P = pistils, and S = stamens. The suffixes −1, −2, and −3 represent three biological replicates (i.e., independent samples) of each tissue and developmental stage. S1 = bud stage, S2 = initial flowering stage, and S3 = full flowering stage.

Transcriptome sequencing analysis during the S1, S2, and S3 stages in S, P, and T organs generated a total of ~1783.08 million reads. Raw read processing and filtering retained on average 97% of the reads, indicating high data integrity. The Q30 score and GC content were 97.27–97.47% and 46.85–48.22%, respectively (Supplementary Table S6). *Overall gene expression* patterns varied between the organs and growth stages (Figure 5A). Correlation analysis indicated consistency among biological replicates (Figure 5B). Among the samples, the PC1 and PC2 samples explained 28.01% and 9.67% of the variability, respectively, indicating clear transcriptional differentiation across the studied organs and developmental stages. Moreover, the distinct clustering patterns suggest stage- and organ-specific variation in gene expression (Figure 5C).

#### 2.6.2. Differential Transcriptome Profiles of *M. cavaleriei* Floral Organs During Development

For the identification of DEGs, genes with a false discovery rate (FDR) < 0.05 and |log_2_FC| ≥ 1 in pairwise comparisons were selected. The number of DEGs varied widely among comparisons, reflecting both stage- and organ-specific transcriptional regulation (Supplementary Figure S1). Generally, the number of DEGs increased with maturity within the same organ when compared across the three stages. The highest number of DEGs was observed in S-S2_vs_P-S2 (35,376) and S-S3_vs_P-S3 (33,898), indicating strong transcriptional differentiation between stamens and pistils. In contrast, within-organ comparisons such as P-S2_vs_P-S1 (1711) and T-S3_vs_T-S2 (2297) showed relatively fewer DEGs, suggesting more gradual stage-dependent changes. These results are consistent with the VOC observations that variability exists at the spatiotemporal level during floral development. K-means clustering of DEGs showed nine distinct expression subclasses. Subclasses such as 1, 4, and 7, containing the largest number of DEGs (7129, 6772, and 15,877, respectively), displayed notable expression peaks at S1 or S3, suggesting stage-specific transcriptional activation. In contrast, clusters like 2 and 6 exhibited overall downregulation patterns from S1 to S3 (Supplementary Figure S2), indicating inactivation of several classes of genes toward floral organ maturity.

##### Transcriptional Changes During Stage Transitioning

**Figure 6:**
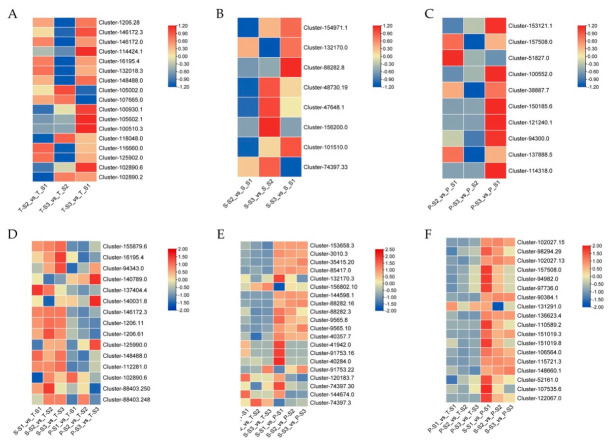
Transcriptional dynamics across floral organs during stage transitioning and organ growth in Tanchun. Differentially expressed gene profiles during stage transitioning (S1-S2-S3) in petals (**A**), stamens (**B**), and pistils (**C**). DEG patterns in an organ-growth-specific manner in petals (**D**), stamens (**E**), and pistils (**F**). Heatmap showing log_2_ foldchange values of key DEG clusters. Each row represents one gene cluster, and each column represents a developmental transition. Color gradients indicate relative expression changes from downregulation (blue) to upregulation (red), scaled by log_2_ foldchange values. T = petals, P = pistils, and S = stamens. The suffixes −1, −2, and −3 represent three biological replicates (i.e., independent samples) of each tissue and developmental stage. S1 = bud stage, S2 = initial flowering stage, and S3 = full flowering stage.

Transcriptome profiling across S1–S3 revealed strong, organ-specific shifts in gene expression, reflecting the progressive functional specialization of floral tissues. Petals showed the most pronounced reprogramming (Figure 6A). The S1 and S2 transition was marked by early activation of precursor-mobilizing and remodeling genes, including multiple AGL (Cluster-1206.28, BGL18-like; Cluster-146172.3 and Cluster-146172.0), chitinase (CHI; Cluster-16195.4 and Cluster-114424.1), endoglucanase-like proteins (EG; Cluster-132018.3), and an amino acid/polyamine transporter (CAT1-like; Cluster-148488.0). In parallel, developmental regulators such as a MADS-box TF (AGL2-like; Cluster-105002.0) and DNA demethylase (Cluster-107665.0) were downregulated, indicating a shift away from organ identity toward metabolic readiness. During S2 and S3, petals strongly induced carbohydrate-mobilizing enzymes, notably TPS/TPP (Cluster-100930.1 and Cluster-105602.1) and oxidative enzymes such as polyphenol oxidase (PPO; Cluster-100,510.3). Regulatory factors, including a homeobox–leucine zipper TF (HD-ZIP; Cluster-118048.0), also had increased expression, coordinating metabolic activation. The expression of structural enzymes, such as pectinesterase (PE; Cluster-116660.0 and Cluster-125902.0), decreased, reflecting the completion of tissue expansion. Across S1 to S3, petals increasingly expressed phenylpropanoid-related CoA-activating enzymes (4CL; Cluster-13066.0) and transporters (AVT1J-like; Cluster-102890), supporting their specialization for metabolite production at bloom.

Stamens followed a distinct trajectory centered on lipid and energy metabolism (Figure 6B). The S1 and S2 transition showed increased expression of acyl-CoA oxidase-like genes (ACX4; Cluster-154971.1), lipoxygenases (9-LOX; Cluster-132170.0), and MEP-pathway homologs (DXS; Cluster-88282.8). During the S2 and S3 transition, stamens showed clear activation of energy-related pathways, reflected by the strong upregulation of ATP synthase subunits (ATPβ; Cluster-47648.1 and Cluster-48730.19) and multiple genes involved in carbohydrate catabolism and mitochondrial energy flow. These included transcripts associated with glycolysis/pyruvate metabolism and electron-transfer flavoprotein-linked oxidation, indicating enhanced respiratory capacity to support rapid pollen maturation. Hormone-responsive genes (TIFY10B; Cluster-156200.0, AUX/IAA12; Cluster-101510.0) were induced. However, terpenoid-synthase-like sequences (GDS-like TPS; Cluster-74397.33) were downregulated, highlighting coordinated hormonal and metabolic regulation.

Pistils exhibited the least dynamic transcriptional changes (Figure 6C). In S1 and S2, modest induction of lipid transfer proteins (HCT; Cluster-153121.1), pectinesterase (PME; Cluster-157508.0), and baseline phenylpropanoid enzymes (PAL; Cluster-51,827.0, CCoAOMT; Cluster-100552.0) signaled gradual preparation for receptivity. The S2 and S3 shift involved selective activation of defense and redox genes, including PR proteins (PR; Cluster-38887.7), peroxidase (PO; Cluster-150185.6 and Cluster-121240.1), and ethylene-responsive TFs (RAP2; Cluster-94300.0, ERF; Cluster-137,888.5; DREB; Cluster-114,318.0), while volatile-related transcription remained largely stable. Overall, pistils maintained a structurally conserved, signaling-oriented expression profile across S1–S3.

Stage-transition transcriptomics revealed that petals undergo the strongest shift from structural remodeling to metabolic activation, stamens predominantly upregulate lipid-, energy-, and terpenoid-related genes, while pistils maintain a conservative profile focused on structural integrity and signaling as the flower progresses from S1 to S3.

##### Transcriptional Changes During Organ Growth

Comparative transcriptome analysis among petals, stamens, and pistils at each developmental stage revealed distinct organ-specific transcript expression trends that remained stable across S1, S2, and S3 (Figure 6). Petals consistently displayed a high expression of genes involved in structural expansion (Figure 6D), precursor release, and volatile readiness. At S1, petals showed strong upregulation of cell-wall remodeling enzymes relative to stamens and pistils, including CHI (Cluster-155879.6, Cluster-16195.4, and Cluster-94343.0) and EG (Cluster-140789.0, Cluster-137404.4, and Cluster-140031.8), along with multiple β-glucosidases (BGL18; Cluster-146172.3, Cluster-1206.11, and Cluster-1206.61), indicating active remodeling and early glycoside hydrolysis during organ enlargement. Transport and metabolic support genes such as an amino acid/polyamine antiporter (CAT1; Cluster-125990.0 Cluster-148488.0) and ABC transporter components (AVT1B; Cluster-112281.0, Cluster-102890.6, Cluster-88403.250, and Cluster-88403.248) were consistently more abundant in petals across all stages, suggesting sustained mobilization of precursors required for later volatile synthesis.

Stamen transcriptomes were dominated by lipid metabolism, energy production, and terpenoid precursor generation (Figure 6E). Across S1, S2, and S3, stamens showed higher expression of fatty acid metabolic genes, including ACX (Cluster-153658.3, Cluster-3010.3, and Cluster-35415.20), lipoxygenases (9-LOX; Cluster-85417.0, Cluster-132170.3, and Cluster-156802.10), and MEP-pathway homologs, particularly 1-deoxy-D-xylulose-5-phosphate synthase (DXS; Cluster-144598.1, Cluster-88282.16, and Cluster-88282.3) and 4-hydroxy-3-methylbut-2-enyl diphosphate reductase (HDR; Cluster-9565.8, Cluster-9565.10, and Cluster-40357.7). Organ growth in stamens was further supported by strong induction of mitochondrial energy-associated genes, including NADH dehydrogenase-related components (NDH; Cluster-41942.0, Cluster-91753.16, Cluster-40284.0, and Cluster-91753.22). Terpenoid synthase-like genes (GDS-like TPS; Cluster-120183.7, Cluster-74397.30, Cluster-144674.0, and Cluster-74,397.3) remained elevated throughout stamen development, aligning with their sustained contribution to terpene biosynthesis.

Pistils showed a somewhat stable transcriptomic profile during growth, with slight activation of structural and signaling pathways but minimal involvement of VOC-related genes (Figure 6F). Across S1–S3, pistils consistently expressed lipid-transfer proteins (LTPs; Cluster-102027.15, Cluster-98294.29, and Cluster-102027.13), pectinesterases (PME; Cluster-157508.0, Cluster-94982.0, and Cluster-97736.0), and baseline phenylpropanoid-pathway enzymes (PAL; Cluster-90384.1, Cluster-131291.0, and Cluster-136623.4), indicating a focus on structural maintenance and reproductive preparation rather than metabolic specialization. Defense and redox-stabilizing genes, including catalase(-like) (Cluster-110589.2, Cluster-151019.3, and Cluster-151019.8), ascorbate peroxidase(-like) (Cluster-106564.0, Cluster-115721.3, and Cluster-148660.1), and pathogenesis-related proteins (PR; Cluster-52161.0, Cluster-107535.6, and Cluster-122067.0), were constitutively expressed, supporting tissue integrity during pistil maturation.

Overall, organ-growth transcriptional signatures showed that petals prioritize structural expansion and precursor mobilization, stamens prioritize lipid and terpenoid metabolic capacity, and pistils maintain a conserved structural and signaling profile, establishing distinct biological trajectories before the onset of full scent production at anthesis.

#### 2.6.3. Pathway-Specific Changes in Gene Expression

The DEG enrichment analysis across floral organs revealed strong stage-dependent modulation of several major metabolic pathways, with petals and stamens showing the highest pathway-level responsiveness and pistils exhibiting more conservative changes (Supplementary Figure S3). During S1 to S2, petals showed broad-scale activation across pathways related to secondary-metabolite biosynthesis, including phenylpropanoid biosynthesis, isoquinoline alkaloid biosynthesis, flavone/flavonoid pathways, and starch/sucrose metabolism. Strong enrichment of cutin, suberine, and wax biosynthesis, beta-alanine metabolism, and amino-sugar/nucleotide-sugar metabolism indicated active structural growth and mobilization of glycosylated precursors. During S2 and S3, petals retained strong enrichment of secondary-metabolite pathways, with notable activation of plant hormone signal transduction, starch/sucrose metabolism, tryptophan and tyrosine pathways, glutathione metabolism, and galactose metabolism. Concurrent enrichment of ribosome and translation-related pathways reflected accelerated metabolic activity as the petals entered full bloom.

Stamens exhibited a more specialized enrichment profile. In S1 to S2, dominant pathways included isoquinoline alkaloid biosynthesis, phenylpropanoid biosynthesis, plant hormone signal transduction, and starch/sucrose metabolism, together with activation of tyrosine, tryptophan, and valine/leucine/isoleucine metabolic branches. Additional enrichment of cutin/wax biosynthesis and glycolysis/gluconeogenesis suggested preparation for pollen development and lipid remodeling. During S2 to S3, stamens showed even stronger enrichment of secondary metabolism, including isoquinoline alkaloids, phenylpropanoids, and glutathione metabolism, supported by pathways related to ribosome function, MAPK signaling, and fatty acid metabolism (including α-linolenic acid). These changes reflect intensified biosynthetic and energy-requiring processes during pollen maturation (Supplementary Figure S3).

Pistils, consistent with their structural and reproductive roles, showed relevant pathway-level dynamics. In S1 to S2, enrichment was observed in secondary-metabolite pathways, phenylpropanoid biosynthesis, starch/sucrose metabolism, cutin/wax biosynthesis, cyanoamino acid metabolism, and amino-sugar/nucleotide-sugar metabolism, indicating gradual cell-wall and surface modifications associated with stigma development. During S2 to S3, pistils activated pathways related to plant hormone signaling, ascorbate/glutathione metabolism, MAPK signaling, cysteine/methionine metabolism, and limited isoquinoline alkaloid biosynthesis. These enrichments reflect redox maintenance, defense readiness, and controlled metabolic adjustments without large shifts in secondary-metabolite production. Across organs, petals and stamens showed the strongest enrichment of pathways associated with secondary metabolism, amino acid metabolism, phenylpropanoid biosynthesis, lipid-related pathways, and starch/sucrose metabolism, especially during the S2 to S3 transition. Pistils exhibited restricted but functionally focused regulation centered on structural maintenance, signaling, and redox balance.

Based on the overall results, below, we specifically highlight the key aroma-related transcriptional changes in petals.

#### 2.6.4. Transcriptomic Changes in Petals

##### Key Transcriptional Changes in Petals During S1 to S2 Transition

**Figure 7:**
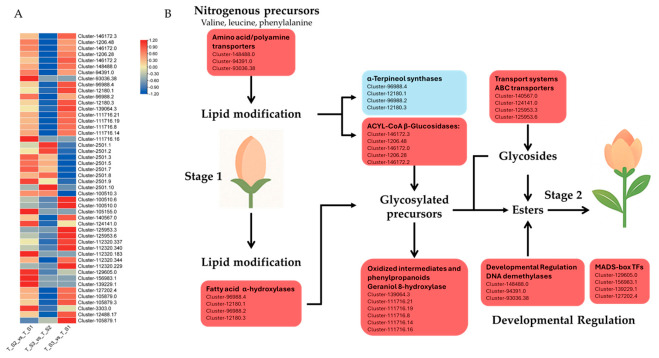
Transcriptional changes associated with the S1 to S2 transition in petals. (**A**) Heatmap showing the normalized expression profiles across three developmental comparisons (T_S2_vs_T_S1, T_S3_vs_T_S2, T_S3_vs_T_S1). Each row represents a specific gene cluster, and each column corresponds to a developmental contrast. The color gradient reflects relative expression levels, with the scale bar indicating standardized expression values. Red indicates higher expression/accumulation, while blue indicates lower expression/accumulation. (**B**) Conceptual model illustrating the principal functional gene groups associated with the S1 (bud stage)–S2 (initial flowering stage) developmental transition. Arrows indicate the inferred directional progression of metabolic and regulatory processes. Colored boxes represent enriched functional gene categories, with cluster IDs corresponding to the differentially expressed genes included within each group. Red and blue indicate upregulation and downregulation, respectively, during the transition.

The developmental transition from S1 (closed bud) to S2 (pre-anthesis) in petals is characterized by major transcriptional reprogramming (Supplementary Table S6). A key VOC-related pathway, i.e., the butanoate metabolism pathway, showed significant changes during this transition (Figure 7). This stage exhibited the large-scale mobilization of metabolic precursors. Multiple transcripts of BGLs (Cluster-146172 and Cluster-1206) were significantly upregulated, suggesting an extensive release of glycosylated volatile precursors and keto acid substrates, which are subsequently used downstream in butanoate and related pathways. Concomitantly, several amino acid/polyamine antiporters such as Cluster-148488.0, Cluster-94391.0, and Cluster-93036.38 were activated, indicating enhanced internal transport of nitrogenous precursors, including valine, leucine, and phenylalanine, which feed directly into acyl-CoA and short-chain acid formation.

Petals also displayed strong induction of lipid-modifying genes critical for supplying medium-chain acyl-CoA pools needed for butanoate metabolism. Multiple fatty acid ω-hydroxylases (FAO-like P450s; Cluster-96988 and Cluster-12180) were upregulated, indicating elevated fatty acid turnover and enhanced β-oxidation activity. The production of these shortened fatty acid intermediates is essential for generating C4–C6 acid backbones that feed directly into butanoate metabolism during later stages.

Notably, during S1 and S2 transition, we observed expression changes indicating rerouting of the metabolic carbon flow away from bulk monoterpene synthesis toward oxidized intermediates and phenylpropanoid precursors, both of which interface with butanoate metabolism. Several geraniol 8-hydroxylase (G8H) transcripts were upregulated (Cluster-139064 and Cluster-111716), while α-terpineol synthase transcripts (Cluster-2501) displayed broad repression. This shift reflects downregulation of non-essential volatile terpene formation in favor of increased flux into phenylpropanoid, benzenoid, and keto acid biosynthesis, consistent with the observed accumulation of butanoate- and phenylalanine-derived precursors in the DAMs dataset. Supporting this redirection, several polyphenol oxidase transcripts (Cluster-100510 and Cluster-105155) were induced, enabling conversion of phenolic intermediates into benzaldehydes and benzoates, which act as upstream substrates for ester formation.

The transcriptome data suggests that, to accommodate the increasing movement and sequestration of specialized metabolites, petals also activated a broad suite of transport systems. Upregulated ABCB/MDR transporters (Cluster-140567.0, Cluster-124141.0, and Cluster-125953) and ABCG/PDR exporters (Cluster-112320) highlighted enhanced vacuolar sequestration and apoplastic export of newly synthesized intermediates. These transport systems are essential not only for metabolite storage but also for preventing cellular toxicity during periods of intense metabolic priming.

Finally, consistent with a shift from developmental identity toward metabolic specialization, several regulatory genes were downregulated. These included MADS-box transcription factors (Cluster-129605.0, Cluster-156983.1, Cluster-139229.1, and Cluster-127,202.4) and DNA demethylase (DML; Cluster-105879.0, Cluster-105879.3, Cluster-3303.0, Cluster-12488.17, and Cluster-105879.1), indicating relaxation of floral organ identity programs and enabling broader activation of secondary metabolism networks.

Taken together, these transcriptional changes show that the S1 and S2 transition is an important stage during which petals activate precursor biosynthesis, enhance acyl-CoA and phenylpropanoid flux, reorganize terpenoid synthesis, and use transport-related pathways. This establishes the biochemical foundation required for the full activation of the butanoate metabolism pathway and the subsequent production of ester-rich floral identity compounds at S3.

##### Key Transcriptional Changes in Petals During S2 to S3 Transition

**Figure 8:**
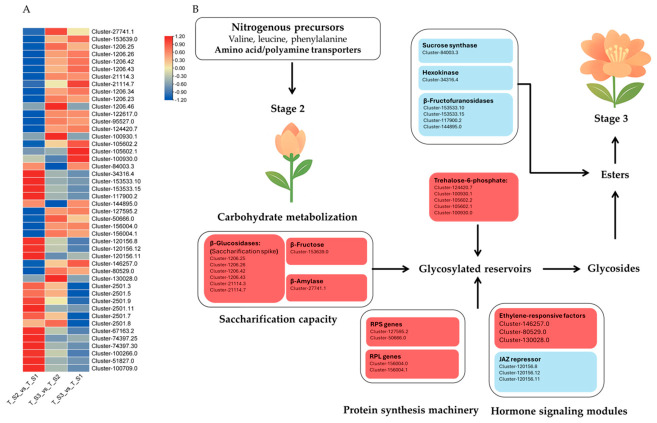
Transcriptional changes associated with the S2 to S3 transition in petals. (**A**) Heatmap shows the normalized expression profiles across three developmental comparisons (T_S2_vs_T_S1, T_S3_vs_T_S2, and T_S3_vs_T_S1). Each row represents a specific gene cluster, and each column corresponds to a developmental contrast. The color gradient reflects relative expression levels, with the scale bar indicating standardized expression values. Red indicates higher expression/accumulation, while blue indicates lower expression/accumulation. (**B**) Conceptual model illustrating the principal functional gene groups associated with the S2 (initial flower stage)–S3 (full flowering stage) developmental transition. Arrows indicate the inferred directional progression of metabolic and regulatory processes during full flower opening and maturation. Colored boxes represent enriched functional gene categories, with cluster IDs corresponding to the differentially expressed genes included within each group. Red and blue denote upregulation and downregulation, respectively, during the S2 and S3 transition.

The transition from S2 to S3 shows the most important transcriptomic and metabolic shift in the petal, marking the key stage at which the organ transitions from the preparatory stage to active biochemical use for pollinator attraction (Figure 8). This stage was characterized by a substantial upregulation of carbohydrate mobilization pathways (Supplementary Table S6). Key members included β-amylase (Cluster-27741.1), β-fructofuranosidase (Cluster-153639.0), and a diverse suite of β-glucosidase isoforms (Cluster-1206, Cluster-21114.3, and Cluster-21114.7). Importantly, this induction showed strong isoform specificity: β-glucosidase 18-like (KO: K05350) and putative β-glucosidase 41 (KO: K01188) were among the most strongly activated, highlighting a targeted remodeling of saccharification capacity. This saccharification wave likely provides (i) rapid access to hexose pools needed to support energy-intensive scent release and (ii) release of glycosylated precursors contributing to downstream volatile production.

Carbohydrate restructuring was further supported by the activation of trehalose-6-phosphate synthase/phosphatase (TPS/TPP; Cluster-124420.7, Cluster-100930.1, Cluster-105602.2, Cluster-105602.1, and Cluster-100930.0). These transcripts suggest crucial involvement of the T6P signaling pathway in balancing carbon utilization with the energetic requirements of scent biosynthesis. Notably, this induction occurred alongside a concerted downregulation of competing carbohydrate-handling isoforms, including sucrose synthase (Cluster-84003.3), hexokinase (Cluster-34316.4), and alternative β-fructofuranosidases (Cluster-153533, Cluster-117900.2, and Cluster-144895.0), demonstrating a tight metabolic prioritization toward high-flux sugar catabolism and volatile-related energy allocation.

This increased metabolic activity was accompanied by an increase in protein synthesis capacity, as shown by the induction of numerous ribosomal protein genes (RP; Cluster-127595.2 and Cluster-50666.0) and ribosomal protein-like genes (RPL; Cluster-156004.0 and Cluster-156004.1). This indicates that at S3, the petals allocate substantial translational resources to constructing and maintaining the enzymatic machinery required for anthesis.

The transcriptome data also suggest the potential role of hormonal regulation in the shift from S2 to S3. JAZ repressors (Cluster-120156) were downregulated, reflecting activation of the jasmonate signaling cascade. We also noted the upregulation of ethylene-responsive transcription factors (ERF; Cluster-146257.0, Cluster-80529.0, and Cluster-130028.0), which might suggest an integration of JA-ET crosstalk in modulating metabolic and developmental transitions.

Overall, the transcriptomic changes in petals during this transition indicate that it did not involve broad activation of volatile biosynthesis pathways that were different from those of the S1 to S2 transition. We observed downregulation of terpenoid synthase genes (TPS; Cluster-2501, Cluster-67163.2, Cluster-74397.25, and Cluster-100266.0) and PAL genes (Cluster-51827.0 and Cluster-100709.0). This repression suggests that S3 petals no longer rely on de novo synthesis of terpene or phenylpropanoid cores, but instead utilize a more energy-efficient strategy. Moreover, the transcriptome results, together with the observations in VOC analysis, show that two complementary mechanisms define this S3 specialization. The first one is the specialized ester production through the butanoate metabolism pathway, as observed in the metabolic enrichment of compounds such as propanoic acid 2-methyl-, 1-methylethyl ester, and ethyl benzoate (Supplementary Table S4). The second mechanism could be the release of a pre-formed volatile reservoir through massive induction of glycoside-cleaving enzymes, including β-glucosidase isoforms (Cluster-1206, Cluster-122617.0, and Cluster-95527.0).

Together, these mechanisms enable the S3 petal to generate a rich, complex, and developmentally timed blend of floral compounds. This transcriptional understanding suggests a highly efficient volatile biosynthesis/emission strategy, integrating rapid precursor mobilization, glycoside hydrolysis, selective ester biosynthesis, and hormone-driven regulatory activation to orchestrate the floral display at anthesis.

#### 2.6.5. Transcription Factor Dynamics Underlying Petal Aroma Programming

The VOC and transcriptome data clearly suggested that the development of a characteristic aroma in the petals is driven by a preparatory stage in the S1 to S2 transition and the activation of highly targeted metabolic pathways from S2 to S3 (Supplementary Table S7). Our results indicate the large-scale role of TFs for VOC biosynthesis during flower transition.

During the S1 to S2 transition (Supplementary Figure S4), petals demonstrated a reduced expression of important TFs, such as the downregulation of JAZ repressors (e.g., Cluster-120156), AUX/IAA repressors (Cluster-111970.0 and Cluster-136708.1), and R2R3-MYB suppressers (e.g., Cluster-109358). These changes suggest the release of early secondary metabolism genes from their repressive effect. Contrarily, multiple ERF TFs (Cluster-103948.0, Cluster-108349.0, Cluster-116782.15, Cluster-122813.0, Cluster-137888.1, Cluster-152782.7, Cluster-53488.5460, Cluster-66296.0, and Cluster-94300.0, Cluster-97722.0), MYC2 (Cluster-6231.6), and WRKYs (Cluster-105733.2, Cluster-105733.3, Cluster-105733.6, Cluster-107397.2, Cluster-11392.7, Cluster-121314.0, Cluster-56181.25, Cluster-61158.0, and Cluster-63106.1) showed increased expressions. These expression changes suggest an early activation of JA-dependent transcriptional networks and the priming of phenylpropanoid-linked and stress-responsive transcriptional elements. Together, these TF expression changes establish the initial level of metabolic activity. It supports the petal for the mobilization of high-flux precursors and the primary hydrolysis of glycosides.

Whereas the VOC and transcriptome data suggested that the S2 to S3 transition indicated a shift to pathway-specific activation. Consistent with these observations, the TF expression trends show higher JA sensitivity and downregulation of JAZ repressors and DELLA (Cluster-107412.2), which impose JA- and GA-mediated restrictions on metabolic activation. Several ERFs (Cluster-130028.0, Cluster-80529.0, and Cluster-81749.3) and bZIP regulators (Cluster-106225.0, Cluster-145835.2, and Cluster-30397.31) were upregulated in S3, compared to S2, suggesting the transcriptional regulation of metabolite release pathways and the availability of sugar. These bZIPs act as integrators of energy-responsive and carbon-status signaling, whereas the expression changes in WRKYs (Cluster-118827.0, Cluster-141563.3, Cluster-149197.3, Cluster-87982.0, and Cluster-91608.0) suggest the probability of the glycoside cleavage organization, phenolic turnover, and stress-linked aroma pathways.

#### 2.6.6. Validation of RNA-Seq Expression Profiles by qRT-PCR

**Figure 9:**
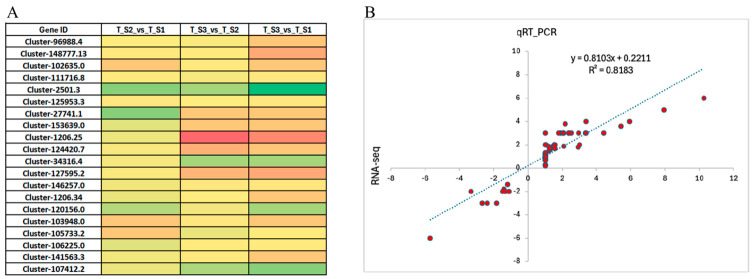
Validation of RNA-seq data by qRT-PCR and correlation analysis: (**A**) Relative expression levels of selected genes involved in floral aroma-related metabolic and regulatory pathways, as determined by qRT-PCR across three developmental stages (S1, S2, and S3). Expression values are shown as the mean ± SD of log_2_ fold change and normalized to the reference gene. (**B**) Pearson correlation analysis between gene expression levels obtained from RNA-seq (log_2_ fold change) and qRT-PCR data for the same set of genes across developmental transitions. Each point represents one gene.

For the validation of RNA-seq-based expression patterns, quantitative real-time PCR (qRT-PCR) analysis was performed. The subset of genes represents key metabolic and regulatory categories involved in floral scent formation. Selected targets included carbohydrate-mobilizing enzymes (β-glucosidase, β-amylase, β-fructofuranosidase, and trehalose-6-phosphate synthase/phosphatase), volatile-related metabolic enzymes (geraniol 8-hydroxylase, α-terpineol synthase, and fatty acid ω-hydroxylase), and transcription factors (ERF, JAZ, bZIP, DELLA, and WRKY) (Figure 9).

A comparison of RNA-seq log_2_ foldchange values with qRT-PCR expression trends demonstrated a strong overall consistency in both directions and relative magnitude of expression changes across developmental transitions (Figure 9A). Genes identified as strongly induced in RNA-seq (including β-fructofuranosidases like Cluster-153639.0 and Cluster-148777.13; β-glucosidases like Cluster-1206.34 and Cluster-1206.25; ribosomal protein genes like Cluster-127595.2; and trehalose-6-phosphate synthase/phosphatase like Cluster-124420.7) showed steady upregulation by qRT-PCR, predominantly during the S2 to S3 transition. This result reinforces the late-stage activation of saccharification and translational capacity. Similarly, the transcriptional repression of DELLA (Cluster-107412.2) and α-terpineol synthase (Cluster-2501.3), detected by RNA-seq, was reproduced by qRT-PCR, supporting the suppression of de novo terpene synthesis and growth-restraining regulators at anthesis.

Quantitative correlation analysis between the qRT-PCR and RNA-seq datasets further endorsed the reliability of the data. Linear regression revealed a strong positive correlation (R^2^ = 0.8183), with a regression equation of *y* = 0.8103*x* + 0.2211 (Figure 9B). This result indicates that estimates of RNA-seq expression accurately reflected the abundance of transcripts measured independently by qRT-PCR. Minor deviations were observed for a small number of low-abundance transcripts, which are consistent with operational sensitivity differences between the two platforms.

## 3. Discussion

Floral fragrance is an important commercial trait in ornamental plants. In recent years, significant progress has been made in understanding the profiles and molecular and regulatory roles of these VOCs. Recent developments in transcriptomics have enabled researchers to identify the global expression patterns of transcripts/genes associated with the differential accumulation of the floral volatiles in organs. Research on the *Michelia* (now merged in the *Magnolia* genus) species, e.g., *M. champaca* [27,28,29], *M. alba* [30,31,32], *M. figo* [33], *M. crassipes* [34], and *M. foveolate* [35], has identified a range of VOCs, indicating that the species in this genus are valuable horticultural and commercial resources. However, despite its value in traditional Chinese culture and horticulture [36], owing to its fragrant white flowers [37], at present, research on *M. cavaleriei*’s floral organs in relation to VOCs and metabolites is limited. In this study, we identified the differential VOC profiles in *M. cavaleriei* petals, stamens, and pistils during three flowering stages: S1–S3. We also combined these results with transcriptome profiles to identify putative transcripts/pathways associated with the observed VOC differences.

The characteristics of floral scents and aroma are determined by the VOC composition and respective content of individual classes and compounds. The differences and abundances of VOCs vary widely in ornamental plant species, creating a diverse range of aromas [38]. Intraspecific differences in VOCs in different cultivars enable characteristic fragrances [39]. The presence of 15 compound classes in three stages of *M. cavaleriei* flowers is so far the most comprehensible list of 1395 compounds, compared to earlier work on *Magnolia* species, which reported 16-67 in *M. champaca* [27,28,29], 61–78 in *M. alba* [30,31,32], and 49 in *M. foveolate* [35]. Our results demonstrate that the major components of *M. cavaleriei* VOCs are terpenoids, esters, nitrogen compounds, hydrocarbons, alcohols, and aldehydes, which is consistent with earlier work. Particularly, earlier studies have reported the presence of phenylpropane ring-types, alcohols, oxygenated sesquiterpenoids, and aliphatic compounds in the volatile oils of *Magnolia* species [27,28,29,30,31,32]. However, it is important to highlight that, based on the comprehensive list identified in our work, a comparison with other species can only be made if a similar methodology is adopted. In terms of the main components, earlier studies on *Magnolia* species have shown that *M. champaca* contains β-linalool and its oxides, methyl benzoate, and phenylethyl alcohol [27,28,29]; *M. alba* volatile oil is majorly composed of dihydrocarveol in buds and linalool in full bloom [30,31,32]; *M. figo* shows isobutyl acetate as a major volatile; *M. crassipes* is rich in α-guaiene, β-caryophyllene, and germacrene B [34]; and *M. foveolata* contains sabinene and terpinen-4-ol in aerial parts, and β-caryophyllene and bicyclogermacrene in leaves [35]. Contrastingly, our results highlight that the most potent odor compound was 3-cyclohexene-1-methanethiol, α,α,4-trimethyl-, which is characterized by a complex sulfury, aromatic, and grapefruit-like odor with naphthyl, resinous, and woody undertones. This was followed by 2,4-Undecadienal, giving green, buttery, and spicy notes, and β-damascone contributing a fruity and floral character along with berry, plum, and tobacco. Among others, butanoic acid, 3-methyl-, 2-phenylethyl ester can also be considered a major odor compound in *M. cavaleriei*, which is characterized by a distinct floral and fruity aroma of rose, peach, and apricot (Supplementary Table S3). These characteristic compounds and respective notes could be a result of multiple factors, including evolution, climate, and growth conditions [40]. However, detailed future analysis might identify the role of each of these factors. Nevertheless, our results conclude that *M. cavaleriei* floral organs are rich in VOCs and impart fragrant notes.

Spatiotemporal variability in the VOC composition and concentration is biologically significant for attracting pollinators toward flowers. In terms of development, flowers produce fragrant compounds upon opening and spreading, as well as at the time of pollination, to harvest maximum pollination benefits [41]. Several studies in different ornamental species, e.g., *Cananga odorata* [42], *Protea* species [43], *Dianthus inoxianus* [44], *Magnolia sirindhorniae* [45], etc., have reported that spatial and temporal patterns of odorant emission from flowers vary [46]. Maximal emission in most species takes place at around the time of flower opening; however, different species exhibit varied rhythms: some have nocturnal maxima, while others emit a peak during the day time [47]. A variety of spatial patterns exist in plants with different organs contributing different compounds of varying amounts [42,43,44,45,46]. Our results are consistent with these studies, both in terms of variation of VOC profiles at different growth stages (S1–S3) as well as different organs. Among the studied *M. cavaleriei* floral organs, petals and stamens had higher differential accumulation, while pistils displayed fewer differential changes. Petals, in flowering plants, are often metabolically active in terms of pigment and VOC synthesis to attract pollinators [41]. Our results that key terpenoids and benzenoids are the major scent-associated compounds in petals are important since emission of these classes of compounds is linked with attraction of pollinators and repulsion of elective visitors in flowering plants [48]. With transition toward maturity, cyclohexanol, 1-methyl-4-(1-methylethenyl)-, benzene–methanol, 3,4-dimethoxy-, phenol, o-amino-, 2,6-octadien-1-ol, 3,7-dimethyl-(Z)-, cyclohexanol, 1-methyl-4-(1-methylethenyl)-, 2,6-octadien-1-ol, 3,7-dimethyl-(Z)-, phenylacetic acid propyl ester and butanoic acid, 2-methylphenyl ester were identified as major marker VOCs in petals. The differential accumulation of these VOCs in petals can be linked with the reprogramming of several metabolic pathways during petal development [49]. Apart from petals, stamens, involved in pollen development, are also metabolically active during flower development [50]. Particularly, the higher number of up-accumulated DAMs in stamens is consistent with their active role in pollen development, which is the key reward to pollinators. Our results are also consistent with the earlier understanding that the transition from closed to open flowers is characterized by a high-energy-level metabolism and transport [51]. The results that pistils, across the three stages, exhibited the least variation, are indicative of the essential and stable role of pistils as the signal of a receptive flower toward specialized pollinators [47]. Moreover, the low number of DAMs may possibly play roles in internal physiological growth and development, including hormone signaling, membrane stabilization, or even in defense responses. Such observations are common in flowering plants, where stable metabolism and specific compound emission in pistils serve a constant and crucial function [52,53,54].

Plant VOCs belong to two distinct groups, i.e., terpenes and terpenoids, and aromatic and aliphatic compounds. The biosynthesis of these compounds involves several pathways, i.e., (mono-, di-, and sesqui/tri-)terpenoid biosynthesis, the terpenoid backbone biosynthesis, the shikimate pathway, and those related to amino acid (several branches) biosynthesis [55,56]. Consistently, our results indicated the enrichment of DAMs and DEGs in these pathways (Figure 4; Supplementary Figure S3). Particularly, the enrichment of an abundant number of DAMs and DEGs enriched in secondary-metabolite biosynthesis, structural growth, and mobilization of glycosylated precursors in the transition from S1 to S2 is biologically relevant. These observations are consistent with the role of these pathways in floral organ development as reported in *Primula* vulgaris [57]. The abundance of terpenes, terpenoids, aromatic, and aliphatic compounds in the floral organs, particularly in petals, indicates that, like other flowering plants [6,48], *M. cavaleriei* petals are the main source of volatiles, although other organs (stamens, pistils, sepals, and nectaries) also contributed to the overall bouquet. These results suggest that the abundance of these compounds during early flower development continues toward full bloom [58]. Consistently, the transition from S2 to S3 was characterized by consistent strong secondary metabolite pathway activation, together with the activation of genes associated with phytohormone signaling, amino acid biosynthesis/metabolism, and sugar metabolism. Moreover, the increased accumulation of phenolic and terpenoid volatiles in S3 stages (mostly in petals) suggests both their role in flower development [59], as well as scent development, match with the pollination time.

The transcriptome data suggests that the regulation of the VOC biosynthesis in *M. cavaleriei* floral organs is complex, whereas the transition from S1 to S2 initiates the increased expression of genes associated with important VOC biosynthesis-related pathways. Notably, the increased expression of AGL, BGL, CHI, EG, and CAT1-like, together with several amino acid antiporters in petals from S1 to S2 transition, suggests the increased focus on the flower development (AGLs and BGLs) [60,61], flavonoid and terpenoid biosynthesis (CHI) [62], increased phytohormone biosynthesis (and signaling; EGs) [63], and transport of amino acids (CAT1-like) [64]. Additionally, our data highlights an increased biosynthesis of phenylpropanoids, benzenoids, keto acid biosynthesis, and polyphenol oxidases during the S1 to S2 transition, suggesting the initiation of crucial functions related to pollinator attraction or defense mechanisms [65]. These observations are further supported by the results on the increased expression of G8Hs and α-terpineol synthase transcripts: the enzymes associated with the terpenoid biosynthesis in plants [66,67]. This increased secondary metabolism during the S1 to S2 transition potentially initiated the activation of transports, e.g., ABCG/PDR, which are known to move increasingly synthesized compounds (e.g., alkaloids and phenolics) across cellular compartments [68,69]. Apart from the increased biosynthesis of VOCs, the S1 and S2 transition was also characterized by the downregulation of flower identity genes like MADS-box [70] and DNA demethylases [71], whereas MADS-box genes (involved in the ABCE model of flower development) usually show higher expression during floral bud development and maintain or reduce their expression during opening [70]. And DNA methylation (including demethylation) is involved in the silencing of MADS-box genes in a stage- and tissue-specific manner [72]. Taken together, the VOC profile and transcriptome data analysis results indicate that the transition from the S1 to S2 stage in *M. cavaleriei* flowers (especially in petals) is characterized by increased phenylpropanoid, terpenoid, acyl-CoA biosynthesis, and transport, as well as a reduction in floral identity-related gene expression.

The transition to a completely open flower mostly occurs when the developmental stages are complete; the aroma and color-related pathways are active and maintain the functions until the completion of the reproductive processes [51,73]. Flower development, particularly toward a completely open flower, is a high-energy transport process [51], which is evident from the increased expression of several carbohydrate mobilization enzyme-related genes, e.g., β-amylase, β-fructofuranosidase, β-glucosidase, β-glucosidase 18-like, etc. This has been reported in other flowering species, e.g., *Rosa damascena*, where significant changes occurred in the transitioning of flowers from stage II to III [74]. The increased activity of these genes, together with the VOC-related pathway genes, was accompanied by increased expression of ribosomal protein(-like) genes, suggesting increased protein synthesis. Development of plant organs, e.g., leaves, in *Arabidopsis*, has been linked to increased expression of ribosomal proteins [75]. Finally, the role of phytohormones in flower development is well established as hormones are principal transducers of genetic information [76]. Particularly, the expression changes in JAZ repressors suggest reception and processing of the JA signal [77]. These observations are also consistent with the role of JA (MeJA) in regulating the volatile synthesis in flowers, e.g., *Chrysanthemum indicum* [78]. Taken together, our data indicates that the transition from S2 to S3 is characterized by the induction of carbohydrate-mobilizing enzymes, regulatory factors, structural enzymes, energy-related pathway genes, and defense and redox genes, as well as genes associated with JA, GA, and ET signaling. These observations are relevant to both flower development and VOC biosynthesis. Integrated metabolomic–transcriptomic approaches have increasingly revealed conserved regulatory frameworks underlying floral scent production in aromatic plants. For instance, coordinated activation of terpenoid and phenylpropanoid pathways during flower opening has been reported in *Rosa laevigata* [79] and *Chrysanthemum indicum* [80], where volatile emission peaks coincide with induction of terpene synthases and jasmonate-responsive regulators. Similarly, in *Hydrangea arborescens* [81] and *Primula forbesii* [82], stage-dependent enrichment of secondary metabolism pathways and carbohydrate mobilization enzymes supports the energetic demand of scent production. Compared with other Magnoliaceae species, such as *Michelia champaca* [29] and *M. alba* [83], our results demonstrate a broader spectrum of VOCs and a strong organ-specific regulatory pattern, particularly in petals. These comparisons suggest that although core biosynthetic pathways are conserved, species-specific regulatory networks likely contribute to the unique aromatic signature of *M. cavaleriei*.

## 4. Materials and Methods

### 4.1. Plant Material

**Figure 10:**
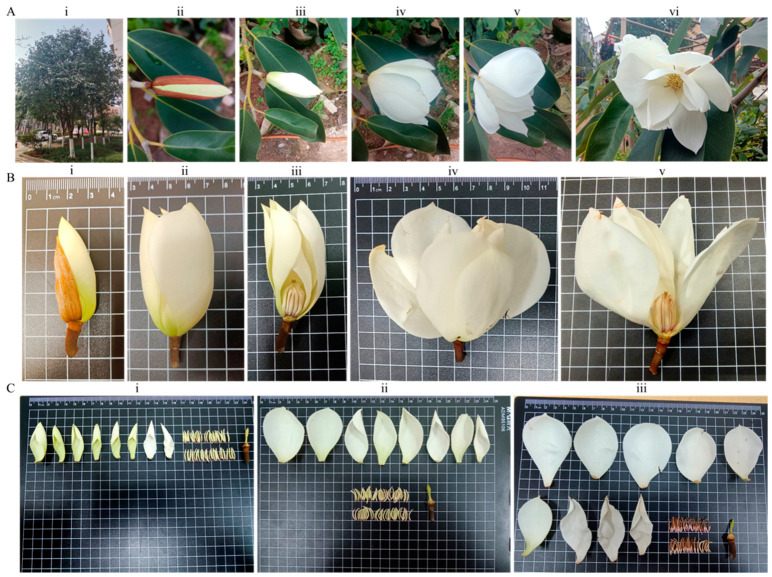
Morphological characteristics of *Magnolia cavaleriei* ‘Tanchun’ floral developmental stages. (**A**) (**i**) represents the crown of the mother tree of ‘Tanchun’ during the flowering season; (**ii**–**vi**) illustrate the overall growth stages of the flower opening. (**B**) (**i**–**v**) show the flowering stages: (**i**) bud stage (S1); (**ii**,**iii**) initial flowering stage (S2); and (**iv**,**v**) full flowering stage (S3). (**C**) (**i**–**iii**) depict the comparison of the three defined floral organs used in this study, i.e., flower petals (T), stamens (S), and pistils (P) at S1 (**i**), S2 (**ii**), and S3 (**iii**).

*Magnolia cavaleriei* var. *platypetala* ‘Tanchun’ is the object of this research. ‘Tanchun’ is a new variety of broad-leaved smiling monkey forest tree that was independently selected by the authors. It was granted a plant variety right (variety right number: 20250312) by the China Forestry and Grassland Administration in July 2025. The samples were collected from the mother plant located at the Central South University of Forestry and Technology during the flowering period of ‘Tanchun’ on 5 February 2025. The collection time was from 9:00 to 11:00 am on sunny days during three developmental stages: S1, S2, and S3. The developmental stages were defined based on clear morphological characteristics as follows: S1 (bud stage): slightly cracked flower buds with partially exposed white petals; S2 (initial opening stage): flower petals slightly unfolded, with stamens still compact and not loosened; and S3 (full blooming stage): fully unfolded petals with a large number of loosened stamens visible (Figure 10). Immediately after harvesting, the flower petals, T, stamens, S, and pistils, P, were quickly separated and placed in 5 mL cryovials. The cryovials were then frozen in a −196 °C liquid nitrogen tank and transported to a laboratory for further analyses.

### 4.2. Qualitative and Quantitative Analysis of Floral Components

#### 4.2.1. Sample Preparation and Gas Chromatography–Mass Spectrometry

Samples were prepared as described in an earlier study by [84]. Briefly, for each stored sample, 500 mg was transferred to a 20 mL headspace vial (Agilent, Palo Alto, CA, USA) containing 2 mL NaCl saturated solution to inhibit any enzyme reaction. The vials were sealed using crimp-top caps with TFE–silicone headspace septa (Agilent). At the time of SPME analysis, each vial was placed in a 60 °C condition for 5 min, and then a SPME Arrow (Agilent) of 120 µm Divinylbenzene/Carbon Wide Range/Polydimethylsiloxane (DVB/CWR/PDMS) was exposed to the headspace of the sample for 15 min at 60 °C.

The desorption of the VOCs from the SPME Arrow coating was carried out in the injection port of the GC apparatus (Model 8890; Agilent) at 250 °C for five minutes. The identification and quantification of VOCs was carried out using an Agilent Model 8890 GC and a 7000D mass spectrometer (Agilent), equipped with a 30 m × 0.25 mm × 0.25 μm DB-5MS (5% phenyl-polymethylsiloxane) capillary column. The carrier gas was helium at a linear velocity of 1.2 mL/min, whereas the injector temperature was 250 °C. The oven temperature was programmed from 40 °C (3.5 min), increasing at 10 °C/min to 100 °C, at 7 °C/min to 180 °C, at 25 °C/min to 280 °C, and then held for 5 min. A series of n-alkane standards (C8–C40) was injected under the same temperature program to calculate retention indices using the van den Dool and Kratz equation. Mass spectra were recorded in electron impact (EI) ionization mode at 70 eV. The quadrupole mass detector, ion source, and transfer line temperatures were set, respectively, at 150, 230, and 280 °C. The MS was selected, and the ion monitoring (SIM) mode was used for the identification and quantification of the analytes. Compound identification was performed using a self-built database at Wuhan MetWare Biotechnology Co., Ltd. (Wuhan, Hubei, China), incorporating retention time, mass spectra matching (one quantitative ion and two to three qualitative ions per compound), and comparison with the literature and authentic standards where available. A compound was positively identified when its retention time matched the standard reference, the calculated retention index fell within ±15 RI units of the NIST value, and all selected ions were present in the sample mass spectrum after background subtraction. During data curation, a small number of detected compounds were identified as non-plant-derived, including potential environmental contaminants or industrial chemicals. These compounds were flagged and removed from the main results and discussion but retained in the supplementary tables for full transparency.

#### 4.2.2. Data Analysis (GC-MS)

Unsupervised principal component analysis (PCA), hierarchical cluster analysis (HCA), and Pearson correlation coefficient (PCC) analysis were performed in R (www.r-project.org; accessed on 7 March 2026). However, before the PCA, the data was log_2_-transformed and mean-centered. The PCC analysis and HCA were presented as heatmaps using the ComplexHeatmap function in R. K-means cluster analysis was performed in R using the base package by unit variance scaling of the GC-MS data.

Differentially accumulated metabolites (DAMs) between the groups were determined by variable importance projection (VIP) > 1 and absolute Log2FC (|Log2FC| ≥ 1.0). The VIP values were extracted from the orthogonal partial least squares discriminant analysis (OPLS-DA) result. The OPLS-DA was performed in R (MetaboAnalystR). The data was log-transformed and mean-centered before OPLS-DA. To avoid overfitting, a permutation test (200 permutations) was performed.

The identified metabolites were annotated using the Kyoto Encyclopedia of Genes and Genomes (KEGG) compound database [85], followed by mapping to KEGG pathways [86].

Sensory flavor annotations (e.g., floral, fruity, spicy, woody, green, and sweet) for each compound were obtained from public databases (e.g., The Good Scents Company http://www.thegoodscentscompany.com, Perflavory http://perflavory.com/; accessed on 15 May 2026, Odour.org http://www.odour.org.uk/odour/index.html; accessed on 15 May 2026, and Food Flavor Lab http://foodflavorlab.cn/; accessed on 15 May 2026). Only compounds with an rOAV value of at least 1 were included in flavor omics analyses (radar charts, flavor wheels, and network diagrams).

### 4.3. Transcriptome Analysis of Floral Components

The collected samples (in triplicate) were used for total RNA isolation by using the RNAprep Pure Plant Kit (Tiangen, Beijing, China). The RNA quality and integrity were determined by a Qubit 4.0 fluorometer (ThermoFisher Scientific Inc., Waltham, MA, USA) and a Qsep400 Bioanalyzer (BiOptic, New Taipei City, Taiwan), respectively. Sequencing libraries were constructed using the NEBNext Ultra RNA Library Prep Kit for Illumina (New England Biolabs, Ipswich, MA, USA). Library quality was tested using the Qubit dye method and a fragment analyzer. The libraries were sequenced on an Illumina platform.

### 4.4. Transcriptome Sequencing Data Analysis

Raw sequencing data was filtered, and the error rate and GC content distribution were determined. Clean reads were spliced using Trinity [87] and used to obtain the reference sequence for subsequent analyses. Assembly integrity was assessed using BUSCO software [88]. The unigenes sequences were aligned with KEGG, NR, Swiss-Prot, GO, COG/KOG, and Temble databases using DIAMOND BLASTX software (v2.2.1) [89]. TransDecoder (https://github.com/TransDecoder/; accessed on 7 March 2026) was used to predict CDS for assembled transcripts. For gene expression quantification (FPKM), the transcripts assembled and made de-redundant by Trinity were used as reference sequences, and the clean reads of each sample were aligned to the reference sequences. This process was performed using Bowtie2 in RSEM [90,91]. The PCC analysis and PCA were computed in R. We used DESeq2 for differential gene identification, and the results were then screened based on |log_2_ Fold Change| >= 1 and FDR < 0.05. The differentially expressed genes (DEGs) were functionally annotated and enriched to KEGG pathways [86] and GO terms [92]. Transcription factors were predicted using the iTAK software [93]. Weighted gene co-expression network analysis (WGCNA) was carried out in R using the WGCNA package.

### 4.5. Quantitative Real-Time PCR (qRT-PCR) Analysis

Using a plant RNA extraction kit and following the manufacturer’s instructions, total RNA was extracted from floral tissues at various stages of development. A spectrophotometer was used to measure RNA concentration and purity. Agarose gel electrophoresis was used to verify RNA integrity. For the following analyses, only high-quality RNA samples were used. According to the manufacturer’s instructions, first-strand cDNA was created from 1 μg of total RNA using a reverse transcription kit and oligo(dT) primers. Quantitative real-time PCR was carried out using a SYBR Green-based master mix on a real-time PCR system. Gene-specific primers, the diluted cDNA template, and the SYBR Green master mix were all included in each 20 μL reaction. The initial denaturation at 95 °C for three minutes, forty cycles of denaturation at 95 °C for ten seconds, and annealing/extension at 60 °C for thirty seconds comprised the thermal cycling conditions. Three biological and three technical replicates were used for each qRT-PCR reaction. The 2^−ΔΔCT^ method was used to calculate relative gene expression levels. Pearson correlation analysis was performed between qRT-PCR and RNA-seq data to validate the reliability of the transcriptome results. The correlation coefficient (R^2^) and regression equation were calculated using Microsoft Excel 2021 Professional Pro.

## 5. Conclusions

*Magnolia cavaleriei* var. *platypetala* ‘Tanchun’ is a newly registered flower variety in China. The analysis of its volatile organic compounds across three developmental stages, i.e., bud stage (S1), semi-opened (S2), and blooming stage (S3), revealed that the major compounds are terpenoids, followed by esters, nitrogen compounds, hydrocarbons, alcohols, and others. The stage transition from S1 to S2 was characterized by green, sweet, fruity, minty, floral, fresh, herbal, cinnamon, cucumber, balsamic, musty, and waxy notes. The S2 to S3 transition was characterized by fruity, sweet, floral, green, oily, coconut, fatty, fresh, alkane, waxy, and creamy notes. The VOCs in Tanchun’s floral organs during transition to full bloom are mostly enriched in mono- and di-terpenoid, secondary metabolite biosynthesis, and phenylalanine metabolism, amino acid (arginine, proline, beta-alanine, glutathione, tryptophan, and tyrosine), butanone, carbon, diterpenoids, glyoxylate, and dicarboxylate metabolism pathways. These observations were also confirmed in the comparative transcriptome analysis of the organs during different stages. The detailed comparative transcriptome analysis of organ-growth showed that petals prioritize structural expansion and precursor mobilization, stamens prioritize lipid and terpenoid metabolic capacity, and pistils maintain a conserved structural and signaling profile, establishing distinct biological trajectories before the onset of full scent production at anthesis. The S1 and S2 transition is an important stage during which petals activate precursor biosynthesis, enhance acyl-CoA and phenylpropanoid flux, reorganize terpenoid synthesis, and use transport-related pathways. This establishes the biochemical foundation required for the full activation of the butanoate metabolism pathway and the subsequent production of ester-rich floral identity compounds at S3. The transition from S2 to S3 involves carbohydrate restructuring, increased protein synthesis, phytohormonal role, and continuation of volatile compound biosynthesis through an energy-efficient strategy. Together, these mechanisms enable the S3 petal to generate a rich, complex, and developmentally timed blend of floral compounds. This transcriptional understanding suggests a highly efficient volatile biosynthesis/emission strategy, integrating rapid precursor mobilization, glycoside hydrolysis, selective ester biosynthesis, and hormone-driven regulatory activation to orchestrate the floral display at anthesis. The detailed list of compounds for each organ at the given stages serves as the foundation for the extraction of specific compounds. Moreover, the respective changes in transcript expression provide a putative list of genes associated with the VOC biosynthetic pathways.

## Figures and Tables

**Table 1:** List of important metabolites accumulated in Tanchun’s floral organs.

Sr No.	Metabolite	Log_2_FC (Max Observed)	Organ/Comparison Where Highest	Chemical Class	Pathway Involvement	Potential Upstream DEGs (Clusters)	Notes/Relevance
1	Benzaldehyde/benzene–acetaldehyde derivatives	2.79–3.3	Multiple	Benzenoid aldehydes	Phenylpropanoid turnover	PAL, PPO, ADH	Sweet floral, almond-like notes
2	Ascaridole	2.49–2.94	Multiple tissues	Monoterpene peroxide	Terpenoid pathway	TPS, CYP monooxygenases	Bioactive monoterpenoid
3	Phenylacetic acid propyl ester	3.20–3.28	Petal and stamen transitions	Benzenoid ester	Phenylpropanoid + AAT	BGLUs, AATs	High-value benzenoid ester
4	Phenol, o-amino-	4.58	Petal S2 vs. S1	Phenolic derivative	Phenylpropanoid/benzenoid	PAL (Cluster-51,827.0, 100,709.0), PPO	Aromatic phenolic precursor
5	2,6-Octadien-1-ol, 3,7-dimethyl-(Z)-	3.67–4.0	Petal S2 vs. S1/stamens	Monoterpenoid	Terpenoid pathway	TPS downregulated clusters	Early terpene-associated shift
6	Benzene–ethanol, α-propyl-	2.89–3.19	Petals and stamens	Aromatic alcohol	Phenylpropanoid derivative	PAL, ADH	Benzenoid aroma component
7	(E)-Hexadec-2-enal	2.01–3.86	Petals and stamens	Fatty acid aldehyde	LOX → ALDH → fatty aldehyde pathway	LOX, ALDH DEGs	Key fatty aldehyde scent component
8	Menthen-4-ol	6.23	Petals S3 vs. S2	Monoterpene alcohol	Terpenoid pathway	TPS (Cluster-2501.xx), CYPs	Strong floral-type monoterpene
9	Butanoic acid, 2-methylphenyl ester	4.36	Petals S3 vs. S2	Ester	Butanoate metabolism (KEGG 00650)	AATs, β-glucosidases (Cluster-1206.xx)	Major fruity/sweet ester contributor
10	Pentanoic acid, ethyl ester	3.83	Petals S3 vs. S2	Ester	Butanoate/fatty acid ester pathway	AAT enzymes	Common floral ester
11	trans-Carveol/cis-Isopiperitenol	3.24	Pistils	Monoterpenoid intermediates	Terpenoid pathway	TPS reductases, CYPs	Oxidized monoterpenoids
12	Nonanoic acid, methyl ester	3.4–3.5	Pistils	Fatty acid ester	Fatty acid → AAT	AAT, LOX	Medium-chain ester aroma
13	2-Pentadecanone	3.24	Pistils	Fatty acid ketone	Fatty acid oxidation	LOX/CYP450	Lipid oxidation marker
14	Lavandulyl caproate	3.39	Pistils	Monoterpene ester	Terpenoid + AAT	AAT	Strong floral ester signature
15	Propanoic acid, 2-phenylethyl ester	3.77	Pistils S3 vs. S1	Benzenoid ester	Phenylpropanoid + AAT	AAT, BGLUs	High-impact floral fragrance ester
16	Cyclohexanol, 1-methyl-4-(1-methylethenyl)-	4.75–4.83	Stamens S3 vs. S2/tissues S3 vs. S1	Terpenoid alcohol	Monoterpene derivative	TPS downregulated, CYP450 oxidases	Marker of oxidative monoterpene turnover

## Data Availability

All data used in this study are available within the text or in the Supplementary Files. The transcriptome data is available at: https://www.ncbi.nlm.nih.gov/bioproject/1380643 (accessed on 28 March 2026).

## Supplementary Materials

The following supporting information can be downloaded at: https://www.mdpi.com/article/10.3390/plants15111646/s1, Supplementary Table S1. List of metabolites detected in *Magnolia cavaleriei* var. *platypetala* ‘Tanchun’. Supplementary Table S2. K-means clustering analysis of Tanchun’s VOCs. Supplementary Table S3. Volatile organic compounds with rOAV values ≥ 1 in at least one sample (*n* = 400). Supplementary Table S4. Differentially accumulated metabolites between Tanchun’s floral organs at different floral growth stages. Supplementary Table S5. List of screened VOCs based on differential accumulation. Supplementary Table S6. List of differentially expressed genes in Tanchun’s petals during floral stage transitions. Supplementary Table S7. Expression trends of differentially expressed transcription factors in Tanchun’s petals during floral stage transitions. Supplementary Figure S1. Transcriptome analysis of *M. cavaleriéi* var. *platypetala* ‘Tanchun’ pistil, stamens, and petals at different developmental stages. S1, S2, and S3 refer to bud, semi-open, and bloom stages, respectively. S, P, and T represent stamen, pistil, and petal, respectively. Supplementary Figure S2. K-means clustering analysis of the differentially expressed genes in Tanchun floral organs during floral stage transitions. Supplementary Figure S3. Enrichment of DEGs in KEGG pathways. The horizontal axis represents the Rich Factor for each pathway, and the vertical axis represents the pathway name (sorted by *p*-value). The color of the dots reflects the *p*-value, with redder dots indicating more significant enrichment. The size of the dots represents the number of differentially expressed genes. Supplementary Figure S4. Heatmap of transcription factor clusters across developmental transitions (S1 → S2, S2 → S3, and S1 → S3). Normalized expression patterns of transcription factor-associated clusters across three developmental comparisons: T_S2 vs. T_S1, T_S3 vs. T_S2, and T_S3 vs. T_S1. Each row represents an individual gene cluster, and each column corresponds to a developmental transition. The color scale (right) reflects relative expression levels, ranging from −1.20 (dark blue, lowest expression) to +1.20 (dark red, highest expression), with intermediate values shown in yellow and light blue. Cluster IDs are listed on the right axis for reference.
