# Supplementary material for: Combined Metabolome and Transcriptome Analysis of Floral Organ Development in Magnolia cavaleriei var. platypetala ‘Tanchun’
Source: Plants (Basel). 2026 May 27;15(11):1646. doi: 10.3390/plants15111646 (PMC13259206; doi:10.3390/plants15111646)
Supplement: Supplementary file 1 [file plants-15-01646-s001.zip › V1 Supplementary Figure S1(1).pptx]

## Slide 1
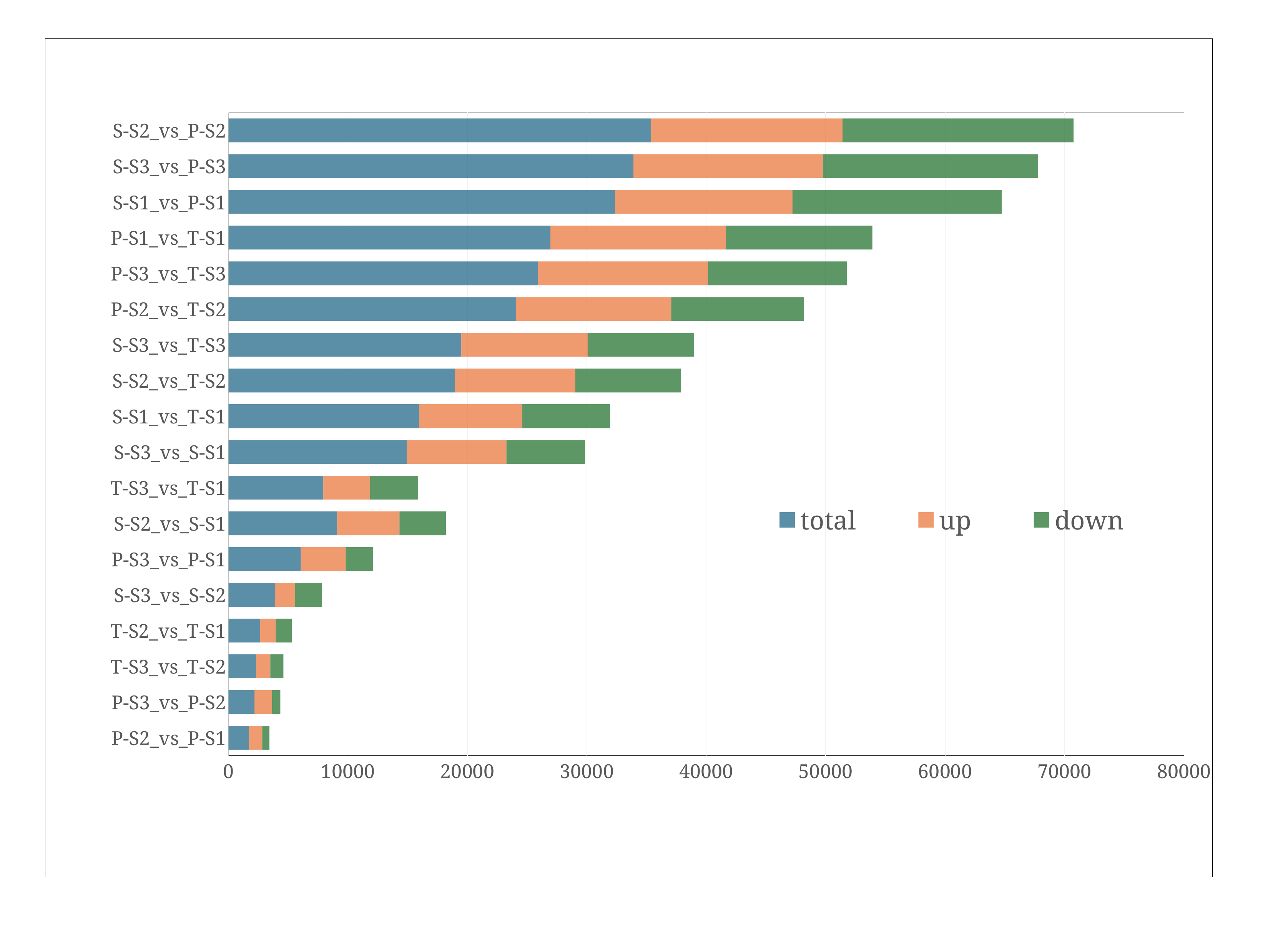

### Chart
| Category | total | up | down |
|---|---|---|---|
| P-S2_vs_P-S1 | 1711.0 | 1109.0 | 602.0 |
| P-S3_vs_P-S2 | 2169.0 | 1479.0 | 690.0 |
| T-S3_vs_T-S2 | 2297.0 | 1200.0 | 1097.0 |
| T-S2_vs_T-S1 | 2648.0 | 1300.0 | 1348.0 |
| S-S3_vs_S-S2 | 3908.0 | 1671.0 | 2237.0 |
| P-S3_vs_P-S1 | 6049.0 | 3757.0 | 2292.0 |
| S-S2_vs_S-S1 | 9100.0 | 5214.0 | 3886.0 |
| T-S3_vs_T-S1 | 7938.0 | 3908.0 | 4030.0 |
| S-S3_vs_S-S1 | 14929.0 | 8336.0 | 6593.0 |
| S-S1_vs_T-S1 | 15966.0 | 8624.0 | 7342.0 |
| S-S2_vs_T-S2 | 18929.0 | 10113.0 | 8816.0 |
| S-S3_vs_T-S3 | 19495.0 | 10572.0 | 8923.0 |
| P-S2_vs_T-S2 | 24086.0 | 12980.0 | 11106.0 |
| P-S3_vs_T-S3 | 25887.0 | 14251.0 | 11636.0 |
| P-S1_vs_T-S1 | 26953.0 | 14677.0 | 12276.0 |
| S-S1_vs_P-S1 | 32362.0 | 14854.0 | 17508.0 |
| S-S3_vs_P-S3 | 33898.0 | 15859.0 | 18039.0 |
| S-S2_vs_P-S2 | 35376.0 | 16032.0 | 19344.0 |
