# Supplementary figures and images for: Combined Metabolome and Transcriptome Analysis of Floral Organ Development in Magnolia cavaleriei var. platypetala ‘Tanchun’
Source: Plants (Basel). 2026 May 27;15(11):1646. doi: 10.3390/plants15111646 (PMC13259206; doi:10.3390/plants15111646)

## Slide 1
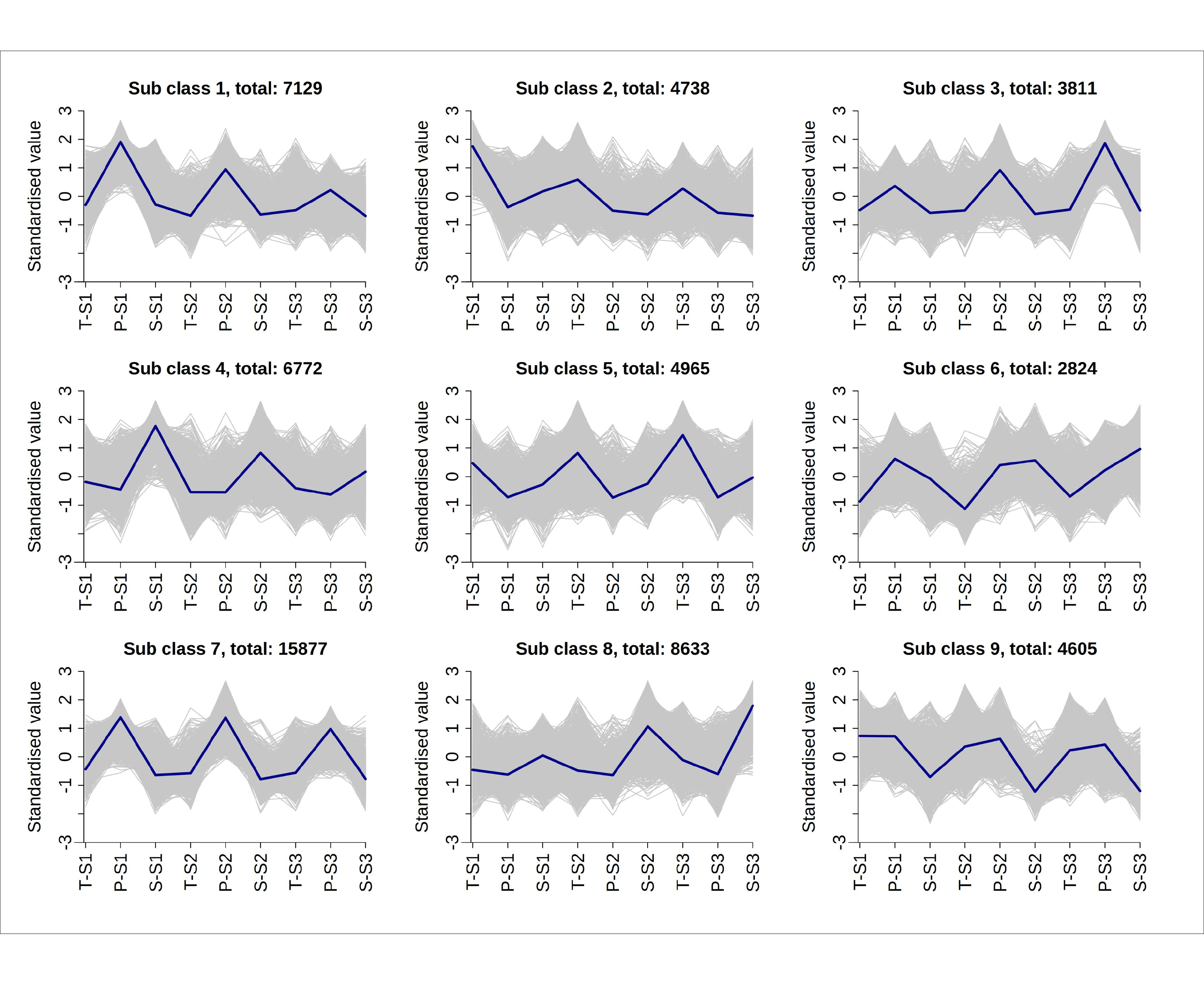

Supplement: Supplementary file 1 [file plants-15-01646-s001.zip › V1 Supplementary Figure S2(1).pptx]

## Slide 1
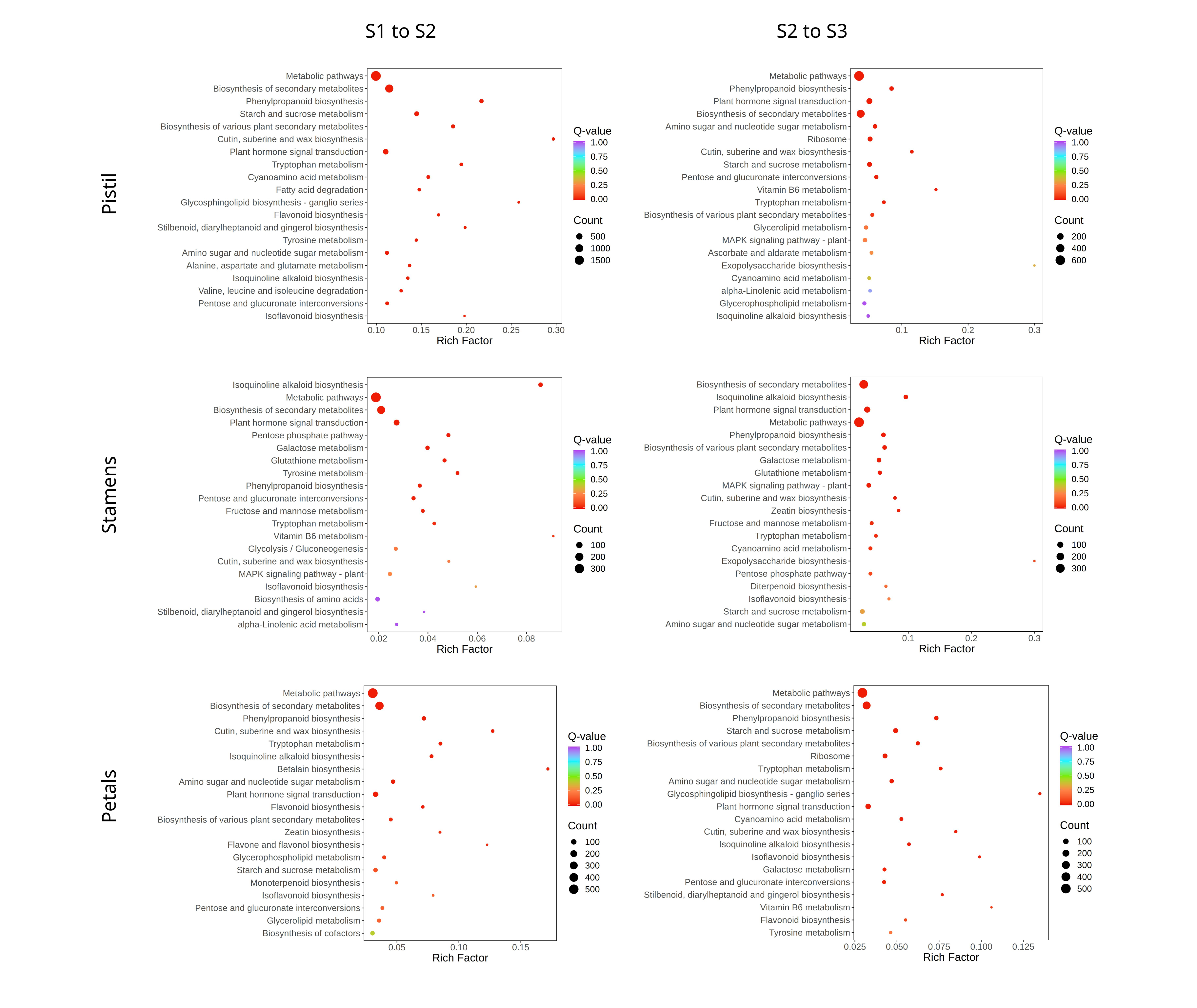

S1 to S2
S2 to S3
Pistil
Stamens
Petals

Supplement: Supplementary file 1 [file plants-15-01646-s001.zip › V1 Supplementary Figure S3(1).pptx]

## Slide 1
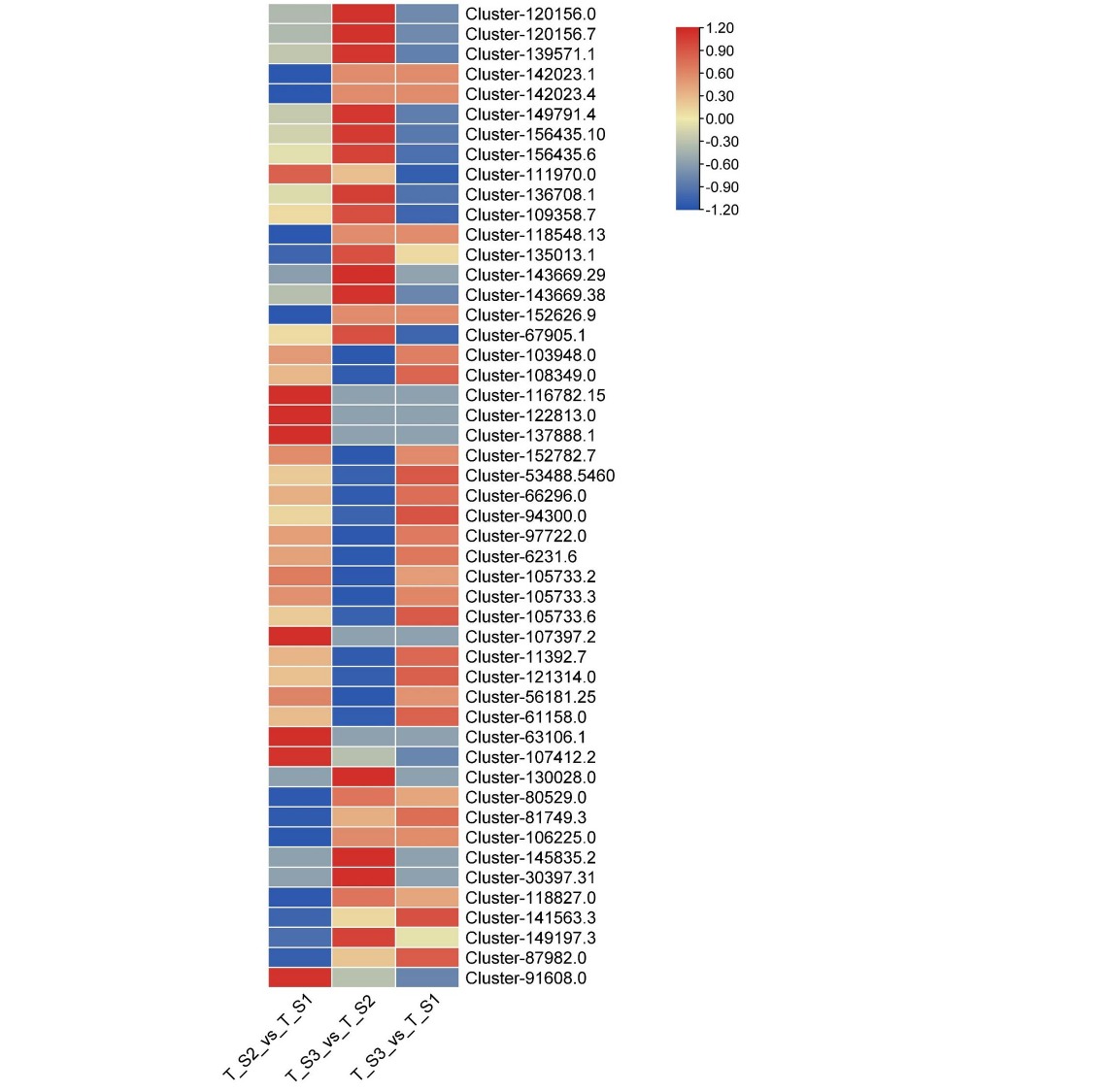

Supplement: Supplementary file 1 [file plants-15-01646-s001.zip › V1 Supplementary Figure S4(1).pptx]
